# Effect of low-volume combined aerobic and resistance high-intensity interval training on vascular health in people with type 2 diabetes: a randomised controlled trial

**DOI:** 10.1007/s00421-024-05473-8

**Published:** 2024-05-02

**Authors:** Emily R. Cox, Trishan Gajanand, Shelley E. Keating, Matthew D. Hordern, Nicola W. Burton, Daniel J. Green, Joyce S. Ramos, Maximiano V. Ramos, Robert G. Fassett, Stephen V. Cox, Jeff S. Coombes, Tom G. Bailey

**Affiliations:** 1 Physiology and Ultrasound Laboratory in Science and Exercise, School of Human Movement and Nutrition Sciences, The University of Queensland, St Lucia, Queensland Australia; 2 Centre for Research on Exercise, Physical Activity and Health, School of Human Movement and Nutrition Sciences, The University of Queensland, St Lucia, Queensland Australia; 3 School of Biomedical Sciences and Pharmacy, University of Newcastle, Callaghan, New South Wales Australia; 4 Active Living and Learning Research Program, Hunter Medical Research Institute, New Lambton, NSW Australia; 5 The Prince Charles Hospital, Queensland Health, Brisbane, Queensland Australia; 6 School of Applied Psychology, Griffith University, Mt Gravatt, Queensland Australia; 7 Menzies Health Institute Queensland, Griffith University, Gold Coast, Queensland Australia; 8 Centre for Mental Health, Griffith University, Brisbane, Queensland Australia; 9 School of Human Sciences (Exercise and Sport Science), The University of Western Australia, Crawley, Western Australia Australia; 10 Caring Futures Institute, College of Nursing and Health Sciences, Flinders University, Adelaide, SA Australia; 11 Institute of Biomedical Technologies, School of Engineering, Auckland University of Technology, Auckland, New Zealand; 12 Princess Alexandra Hospital, Woolloongabba, QLD Australia

**Keywords:** Blood pressure, Diabetes mellitus, type 2, Exercise, Flow-mediated dilation, High-intensity interval training, Pulse wave velocity

## Abstract

**Purpose:**

We compared the effects of low-volume combined aerobic and resistance high-intensity interval training (C-HIIT), combined moderate-intensity continuous training (C-MICT) and waitlist control (CON) on vascular health after 8-weeks of supervised training, and an additional 10-months of self-directed training, in adults with type 2 diabetes (T2D).

**Methods:**

Sixty-nine low active adults with T2D were randomised to 8-weeks of supervised C-HIIT (3 times/week, 78-min/week), C-MICT (current exercise guidelines, 4 times/week, 210-min/week) or CON. CON underwent usual care for 8-weeks before being re-randomised to C-HIIT or C-MICT. This was followed by 10-months of self-directed training for participants in C-HIIT and C-MICT. Vascular outcomes were evaluated at baseline, 8-weeks, and 12-months.

**Results:**

After 8-weeks, supervised C-HIIT significantly improved relative flow-mediated dilation (FMD) compared with CON (mean difference [MD] 0.8% [0.1, 1.4], *p* = 0.025). Although not significantly different from CON, the magnitude of change in relative FMD following 8-weeks of supervised C-MICT was similar (MD 0.8% [–0.1, 1.7], *p* = 0.080). There were no differences in haemodynamic indices, carotid-femoral pulse wave velocity (cfPWV), or aortic reservoir pressure between groups at 8-weeks. After 12-months, there was a significant reduction in haemodynamic indices (time effect, *p* < 0.05) for both C-HIIT and C-MICT, with no between-group difference. The reduction in cfPWV over 12-months was significantly greater in C-MICT than C-HIIT (group × time effect, *p* = 0.018). There was no difference in FMD over time or between groups at 12-months.

**Conclusions:**

Short-term supervised C-HIIT and C-MICT both increased brachial artery FMD compared with CON. Long-term C-HIIT and C-MICT were beneficial for improving haemodynamic indices, but not brachial artery FMD. C-MICT was superior to C-HIIT for improving cfPWV at 12-months.

**Trial Registration:** Australian New Zealand Clinical Trials Registry Identifier ACTRN12615000475549.

**Supplementary Information:**

The online version contains supplementary material available at 10.1007/s00421-024-05473-8.

## Introduction

People with type 2 diabetes (T2D) are at a 2- to 5-fold greater risk of cardiovascular disease and associated events than the general population (Hadi and Suwaidi 2007). Haemodynamic indices including brachial and central blood pressure, arterial stiffness, and vascular endothelial dysfunction are early indicators of atherosclerotic disease (Thijssen et al. 2011; Hametner et al. 2014). Such markers provide pre-clinical targets for lifestyle interventions aimed at reducing cardiovascular risk (Cox et al. 2022).

Exercise is an important lifestyle strategy for the management of T2D. Yet, exercise participation remains low in people with T2D (Nolan et al. 2016), with lack of time a common self-reported barrier (Thomas et al. 2004). This has prompted the prescription of high-intensity interval training (HIIT) in people with T2D as it provides equal or superior metabolic benefits to moderate intensity continuous training (MICT) (Gibala and McGee 2008), in a time-efficient manner. Compared with MICT and/or usual care, high-volume (≥ 15 min at high-intensity) aerobic-only HIIT induces comparable and/or superior benefits for vascular health (Ramos et al. 2015; Way et al. 2019). A substantially lower volume of aerobic-only HIIT (36 min/week, including warm-up/cool-down) induces similar improvements as high-volume HIIT (105 min/week, including warm-up/cool-down) and/or traditional MICT (150–210 min/week) in arterial stiffness (Way 2020), flow-mediated dilation (FMD) (Ghardashi Afousi et al. 2018), and aortic reservoir pressure (ARP) (Ramos et al. 2016) in people with metabolic disease. It has been postulated that higher intensity exercise stimulates greater blood flow through the vessels supplying oxygen to the working muscles, ultimately promoting greater shear stress-induced nitric oxide bioavailability, thus improving vascular health (Thijssen et al. 2009a). Other potential mechanisms for the superior effect of HIIT versus MICT include attenuation of traditional cardiovascular risk factors, insulin resistance, oxidative stress, and inflammation (Ramos et al. 2015).

The current exercise guidelines for people with T2D recommend a combination of aerobic and resistance training given the differential benefits each modality provides for glycaemic control (Hordern et al. 2012). However, studies investigating the effect of exercise on vascular health have typically focussed on aerobic-only training, with limited evidence for the benefits of combined aerobic and resistance exercise training in this population. A systematic review and meta-analysis of 15 studies up to March 2015 reported only three studies of combined aerobic and resistance training on arterial stiffness in people with T2D and indicated no benefit (Way et al. 2016). Since then, Magalhaes et al. (2019) reported improvements in carotid intima media thickness and peripheral arterial stiffness after 12-months of supervised high-volume aerobic HIIT combined with resistance training. No published study to date has investigated the short- and long-term effect of both combined aerobic and resistance exercise training, and the influence of exercise intensity, on markers of vascular health in people with T2D.

As part of the Exercise for Type 2 Diabetes (E4D) Trial, we compared the effects of 78 min/week of low-volume, combined aerobic and resistance high-intensity interval training (C-HIIT) with the current exercise guidelines for people with T2D of 210 min/week of combined aerobic and resistance moderate intensity continuous training (C-MICT) and waitlist control (CON) on haemodynamic indices, arterial stiffness, and brachial artery FMD in people T2D, after 8-weeks of supervised training (phase one). The second aim was to investigate the effectiveness of 12-months (8-weeks of supervised training and 10-months of self-directed training) of C-HIIT and C-MICT on these outcomes (phase two). We hypothesized that both C-HIIT and C-MICT would be superior to CON for improving vascular health after 8-weeks of supervised training. Furthermore, 12-months of C-HIIT and C-MICT would be comparable for improvements in vascular health in people with T2D.

## Research design and methods

This randomised controlled trial was part of the “Exercise for Type 2 Diabetes (E4D)” Trial (Australian New Zealand Clinical Trials Registry ACTRN12615000475549), which investigated the short- and long-term efficacy, safety and feasibility of low-volume combined aerobic and resistance high-intensity interval training in people with T2D. The E4D Trial was prospectively approved by The University of Queensland Human Research Ethics Committee (ethics approval number 2015000164), and adhered to the Declaration of Helsinki principles. This manuscript will focus on predefined secondary outcomes of vascular health, including haemodynamic indices, ARP, arterial stiffness, and brachial artery FMD.

### Participants

Recruitment occurred from October 2015 to November 2018. Participants were eligible for the E4D Trial if they were aged 18–80 years with a diagnosis of T2D, including a glycated haemoglobin (HbA_1c_) of  ≥ 6.0%. The exclusion criteria were per the American College of Sports Medicine’s absolute contraindications to exercise including unstable angina, recent myocardial infarction, coronary artery disease, and uncontrolled, symptomatic heart failure. Potential participants were excluded if they self-reported more than 150 min of moderate physical activity, or 75 min of vigorous physical activity, or any equivalent combination, per week. Written informed consent was obtained at enrolment. Following completion of baseline testing, participants were randomised 1:1:1 to C-HIIT, C-MICT, or waitlist CON. This was stratified based on age and gender. Participants in the waitlist CON group were re-randomised 1:1 to either C-HIIT or C-MICT after 8-weeks using the same procedure. A member of the research team not directly associated with the study completed randomisation and allocation, using a computer-generated sequence.

### Trial design

**Fig. 1:**
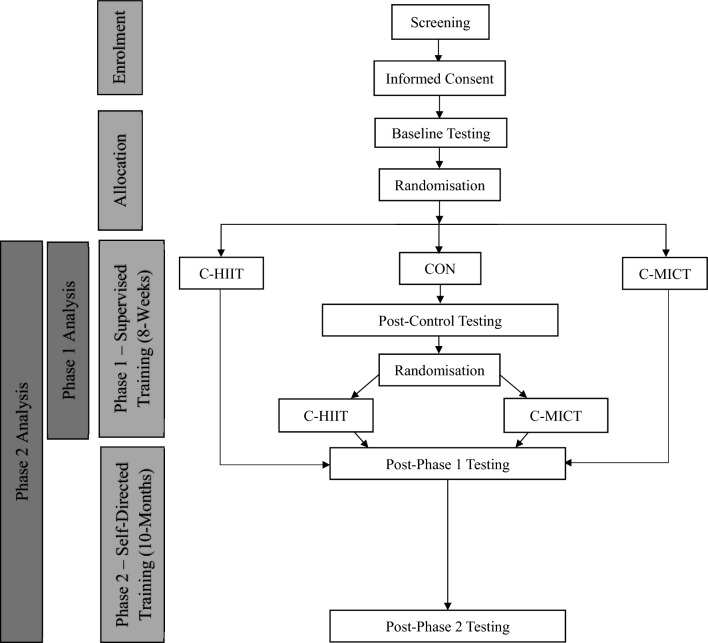
Flow diagram for enrolment, group allocation and phases one and two in the E4D Trial. *C-HIIT* Combined High-Intensity Interval Training; *C-MICT* Combined Moderate Intensity Continuous Training; *CON* Waitlist Control

The trial involved two phases (Fig. 1); phase one involved 8-weeks of supervised exercise training, and phase two involved 10-months of self-directed exercise training, for a total intervention duration of 12-months. Those initially randomised to waitlist CON underwent 8-weeks of usual care, before being re-randomised to either C-HIIT or C-MICT. Participants in CON were re-randomised after phase one as it was considered ethical to provide all trial participants with access to the “intervention”, as well as to maintain participant engagement in the trial. Testing occurred at baseline and after 8-weeks for the three groups (C-HIIT, C-MICT, CON), and again after 12-months for the two exercise groups (C-HIIT, C-MICT). Vascular outcomes included haemodynamic indices, ARP, arterial stiffness, and brachial artery FMD at baseline, 8-weeks, and 12-months.

### Interventions

#### Phase one–supervised exercise training (8 weeks)

Exercise training for both C-HIIT and C-MICT was conducted at The University of Queensland. The Accredited Exercise Physiologist (AEP) to participant ratio was a maximum of 1:2. The mode of aerobic exercise (treadmill, upright bike, recumbent bike) was determined by an AEP before the baseline cardiopulmonary exercise test (CPET), based on the presence of orthopaedic limitations. The resistance-based exercises involved a combination of machine-based, bodyweight, and free-weight exercises. The order the resistance exercises were completed was: (1) leg press, (2) chest press, (3) leg press repeated, (4) seated row, (5) calf raises, (6) shoulder press, (7) abdominal crunch, and (8) bicep curls. During the supervised sessions, workload, number of repetitions during resistance training, heart rate (HR) and rating of perceived exertion (RPE; BORG 6–20 scale) were recorded by the AEP for all participants to monitor adherence to the exercise protocol.

The **C-HIIT group** trained for 26 min, three times per week (78 min/week), on non-consecutive days. Each session began with an aerobic warm-up for 3 min at 50–60% of HR peak (HR_peak_; determined from the peak HR achieved during the CPET) before 4 min of high-intensity aerobic exercise at 85–95% of HR_peak_. The goal was to reach the target zone within the first 2 min. Following 1-min of rest, participants completed 8 × 1-min intervals of high-intensity resistance exercise at an RPE of  ≥ 17 (very hard); each of the eight exercises listed above were completed for 1-min. Participants completed as many repetitions (at least five; aiming for 10–25) as possible within each 1-min bout while maintaining correct technique. One minute of rest separated each interval. The session ended with an aerobic cool-down for 3 min at 50–60% of HR_peak_. This strength-endurance approach was chosen to reflect a typical cardio-based HIIT prescription.

The **C-MICT group** trained for 52.5 min four times per week (210 min/week) – two sessions incorporating both aerobic and resistance training, and two sessions involving aerobic training only. These four sessions alternated during the week. For the two combination sessions, the participants completed 22 min and 30 s of aerobic exercise at 55–69% of HR_peak_ followed by 30 min of resistance-based exercises at a moderate intensity (RPE 11–13; fairly light to somewhat hard). Each resistance-based exercise consisted of two sets of 10 repetitions, with each set separated by a 1-min rest period. The resistance-based exercises completed were identical to C-HIIT (listed above). For the two aerobic-only sessions, participants completed 52 min and 30 s of aerobic exercise at 55–69% of HR_peak_. This C-MICT program is comparable with the current exercise recommendations by Exercise and Sports Science Australia for people with T2D (Hordern et al. 2012). All participants were able to complete the intervention as prescribed (i.e., in a single bout).

The **waitlist CON group** continued with usual care and were asked to maintain their usual physical activity and dietary habits.

#### Phase two–self-directed training (10-months)

Following the supervised exercise phase, all participants commenced phase two of the program, which involved 10-months of self-directed exercise training. Participants were given a logbook containing information about exercises they could complete at home, with their bodyweight or equipment that was readily available to them, that attempted to replicate their C-HIIT/C-MICT supervised training program. They were asked to complete training logs to aid tracking of their adherence, including providing information about intensity via HR (where participants owned a device with HR monitoring capacity) or RPE. In addition, participants were offered optional, once monthly supervised exercise training sessions at the University that was identical to their allocated group, to support their progress.

### Outcomes

All vascular health measurements were completed after an overnight fast (≥ 12 h), and at least 24 h with no exercise, caffeine, alcohol, and tobacco, in accordance with guidelines (Thijssen et al. 2011). Participants were instructed to maintain their normal medication regimen during the preceding 24 h. Participants rested quietly, supine in a dimly lit, temperature-controlled room (24 ± 2 °C) for 15 min prior to assessment.

#### Haemodynamic indices, aortic reservoir pressure (ARP) and arterial stiffness

Indirect assessment of haemodynamic indices, ARP, and arterial stiffness were completed using a SphygmoCor® XCEL (AtCor Medical Pty Ltd., Sydney, Australia). For pulse wave analysis (PWA), the brachial cuff was placed around the brachial artery of the right arm, between the elbow and shoulder. PWA measured brachial systolic (bSBP) and diastolic (bDBP) blood pressures, as well as mean arterial pressure (MAP), central haemodynamics including systolic pressure (cSBP), diastolic pressure (cDBP), pulse pressure (cPP), and augmentation index (AIx). Due to the influence of resting heart rate (HR) (Wilkinson et al. 2000), AIx was adjusted for a HR of 75 beats per minute (AIx@75). Wave separation analysis was completed using SphygmoCor® XCEL software (Version 1.3; AtCor Medical Pty Ltd., Sydney, Australia). This method assumes a triangular-shaped flow wave approximated from the estimated aortic pressure wave (Westerhof and Westerhof 2013). The forward (Pf) and reflected (Pb) pressure waves correspond to the peak and the end of the assumed flow wave, respectively. The reflection magnitude was calculated as Pb/Pf × 100 (presented as a %). The central pressure waveform generated was used to calculate the amplitude and the area under the curve of the ARP using custom-written MATLAB algorithms (version R2017b, MathWorks, Inc., Natick, MA, USA) (Ramos et al. 2016). ARP describes the function of the aorta to temporarily store blood and attenuate pulsatile pressure during ventricular ejection (via the reservoir function) before the aorta recoils during cardiac relaxation and the stored blood is discharged. PWA was assessed three times with the first measure discarded and the average of the second and third measures used for analysis. Additional assessments of PWA were taken if the coefficient of variation for bSBP was greater than 15% between the second and third measures.

Carotid-to-femoral pulse wave velocity (cfPWV) was used as the gold-standard assessment of arterial stiffness. For cfPWV, a cuff was placed around the mid-thigh and a tonometer pressure sensor on the carotid artery, to simultaneously capture the pulse waveforms at femoral and carotid sites. The velocity of pulse transfer from the carotid artery to the femoral artery was measured (using the subtraction method) and calculated according to standardised guidelines (Townsend et al. 2015). An average of the first two consecutive measurements of cfPWV was used for analysis. However, in line with published guidelines (Townsend et al. 2015), cfPWV was repeated if the difference between the two measures of cfPWV was greater than 0.5 m·s^–1^, with the median value used for analysis.

#### Vascular function

Brachial artery FMD was used as an index of vascular function. FMD was assessed using high-resolution Doppler ultrasound (uSmart 3300, Teratech Corporation, Burlington, USA), as per published guidelines (Thijssen et al. 2011). B-mode images of the brachial artery in the distal third of the right upper arm (proximal to the antecubital fossa) were captured using a 7.5 Hz probe. The probe was orientated to the longitudinal plane. Following image optimisation, continuous images were recorded for 1-min to measure baseline artery diameter and blood velocity, using an insonation angle of  ≤ 60°. A forearm cuff placed distal to the olecranon process was then inflated to 220 mmHg, or 50 mmHg above systolic pressure. The cuff was inflated for 5 min to produce forearm ischaemia. Upon cuff deflation, continuous recording occurred for 3 min. Briefly, from recordings of the synchronised artery diameter and blood velocity data, blood flow (the product of lumen cross-sectional area and Doppler velocity) was calculated at 30 Hz. Shear rate (an estimate of shear stress independent of viscosity) was calculated as four times the mean blood velocity/vessel diameter (Black et al. 2008). All recordings were analysed using specialised, automated, edge-detection and wall-tracking software, to provide an objective measure of peak artery diameter and shear rate area under the curve (Woodman, et al. 1985). All recordings were blinded for analysis.

#### Glycaemic control, body mass and composition, and cardiorespiratory fitness

A fasting venous blood sample was taken, with HbA_1c_ and fasting blood glucose assessed using manufacturer supplied assay kits in an automated analyser (Randox RX daytona + , Kearneysville, WV, USA).

Body mass was measured using floor scales (AWB120, Avery Weigh-Tronix Bench, Egham, Surrey, UK). Body fat was determined using Dual-energy X-ray absorptiometry (Discovery, Hologic Inc., Bedford, MA, USA).

Participants completed a graded CPET to determine peak oxygen uptake ($\dot{{\text{V}}}$O_2peak_). Pulmonary gas exchange was assessed using either a Parvo (*n* = 67; Parvo Medics TrueOne, Sandy, UT, USA) or Metamax (*n* = 2; Metamax II system, Cortex, Leipzig, Germany) metabolic system. The metabolic system and exercise mode used at each assessment (i.e., baseline, 8-weeks, and 12-months) was identical for each participant. $\dot{{\text{V}}}$O_2peak_ was assessed as the mean of the two highest 10-sec epoch values attained during the test (where the difference in $\dot{{\text{V}}}$˙O_2_ between values was no greater than 150 mL/min).

### Intervention attendance and adherence

Session attendance and exercise intensity adherence for C-HIIT and C-MICT participants were determined by a combination of AEP-reported exercise records (phase one) and self-report exercise logs (phase two). Attendance was defined as the number of exercise sessions completed as a function of total prescribed sessions. Adherence to the allocated exercise intensity was determined from HR and RPE achieved during exercise sessions.

### Statistical analysis

The sample size for the predefined secondary outcomes (including haemodynamic indices, ARP, cfPWV, and FMD) was based on the predefined primary outcome of the E4D Trial, HbA_1c_ (%). In brief, we determined that 66 participants (22 each in C-HIIT, C-MICT, and CON) would be sufficient to detect a clinically significant (0.6%) difference in HbA_1c_ between groups (Lenters-Westra et al. 2011), with an SD of 2.4%, power of 0.8, and 0.05 significance level. Reductions in HbA_1c_ of this magnitude have been shown in meta-analyses investigating exercise interventions in people with T2D (Mello et al. 2021; Jang et al. 2019).

For these secondary outcomes of the E4D Trial, statistical analyses were completed using SPSS version 27 for Windows (IBM, Armonk, USA). The Shapiro–Wilk test and visual inspection of the distribution of models’ residuals were used to test normality; no transformations were required. The significance level was set at *p* < 0.05 for all analyses. Adjustment for multiple comparisons was made using the Bonferroni approach.

Given the influence of baseline diameter and age on FMD% (Holder et al. 2021), these parameters were included as covariates in the analysis. FMD was not normalised for shear rate area under the curve (SR_AUC_) as previous studies have shown a weak correlation between this and the FMD response in adults ≥ 50 years (Thijssen et al. 2009b).

#### Aim 1–phase one (8-weeks of supervised training)

To determine changes in markers of vascular health after phase one (baseline to 8-weeks), comparisons between C-HIIT and CON, C-MICT and CON, and C-HIIT and C-MICT were completed using intention-to-treat analysis of covariance (ANCOVA), adjusting for baseline values (Figure S1), with Bonferroni post hoc analysis. The change score (baseline to 8-weeks) was used as the dependent variable. Group mean change scores were imputed for dropouts and missing data (Armijo-Olivo et al. 2009).

#### Aim 2–phase two (8-weeks of supervised training and 10-months of self-directed training)

To determine changes in markers of vascular health after phase two (baseline to 12-months), an intention-to-treat analysis with linear mixed modelling was used with the participants as random factors, and the exercise groups (C-HIIT, C-MICT) and time points (baseline, 12-months) as fixed factors. This analysis included waitlist CON participants who had been re-randomised to either C-HIIT or C-MICT, which included 8-weeks of supervised training and 10-months of self-directed training (Figure S2). Missing data were accounted for using maximum likelihood estimation on all available data, with Bonferroni adjustment for multiple comparisons.

For both phases, sensitivity analyses were completed, including a per-protocol analysis excluding those who did not attend *and* adhere to the prescribed intensity during at least 70% (phase one) or 50% (phase two) of the intervention, and an intention-to-treat analysis to account for cardiac medication (anti-hypertensives, statins) changes during the intervention period. These data are presented as Supplementary Material (see Tables S1–S4).

## Results

### Participant characteristics

Four hundred and forty-five individuals were assessed for eligibility for the E4D Trial (Figure S1). Sixty-nine individuals with T2D (mean age 59.5 ± 8.8 years, males 61%, HbA_1c_ 8.5 ± 1.8%) were included in this analysis (Figures S1 and S2). The participant characteristics at baseline are shown in Table 1. Based on recently published reference values (Holder et al. 2021; The Reference Values for Arterial Stiffness Collaboration 2010), on average, the participants had elevated arterial stiffness (cfPWV; 9.2 ± 1.6 m·s^–1^) and impaired vascular function (FMD%; 3.8 ± 1.9%) at baseline.

**Table 1:** Participant Characteristics

	**All**	**C-HIIT**	**C-MICT**	**CON**
**Variable**	*n* = 69	*n* = 23	*n* = 23	*n* = 23
Female, *n* (%)	27 (39.1)	9 (39.1)	9 (39.1)	9 (39.1)
Age (years)	59.5 ± 8.8	59.0 ± 8.8	60.1 ± 7.3	59.4 ± 10.2
Duration of Diabetes (years)	10.4 ± 7.8	9.2 ± 7.4	10.6 ± 8.4	11.3 ± 7.3
HbA_1c_ (%)	8.5 ± 1.8	8.6 ± 2.1	9.0 ± 1.8	8.1 ± 1.3
HbA_1c_ (mmol·mol)	70 ± 20	70 ± 24	75 ± 20	65 ± 14
FPG (mmol·L)	8.9 ± 2.8	9.0 ± 2.8	9.1 ± 2.8	8.6 ± 2.6
Total cholesterol (mmol·L^–1^)	4.1 ± 0.9	4.0 ± 0.8	4.4 ± 1.0	4.0 ± 0.8
LDL-C (mmol·L^–1^)	2.5 ± 0.9	2.5 ± 0.9	2.8 ± 1.0	2.2 ± 0.8
HDL-C (mmol·L^–1^)	1.2 ± 0.3	1.2 ± 0.4	1.2 ± 0.3	1.2 ± 0.3
Triglycerides (mmol·L^–1^)	1.6 ± 0.8	1.6 ± 0.8	1.6 ± 0.9	1.6 ± 0.8
BMI (kg·m^–2^)	33.5 ± 6.1	32.6 ± 5.1	34.0 ± 6.6	33.9 ± 6.4
$\dot{{\text{V}}}$O_2peak_ (mL·kg^−1^·min^−1^)	24.1 ± 5.8	24.3 ± 5.1	24.7 ± 7.0	23.3 ± 5.3
**Medications**				
Oral Anti-hyperglycaemics, *n* (%)	61 (88.4)	20 (87.0)	20 (87.0)	21 (91.3)
Insulin, *n* (%)	15 (21.7)	4 (17.4)	3 (13.0)	8 (34.8)
Anti-hypertensives, *n* (%)	51 (73.9)	17 (73.9)	20 (87.0)	14 (60.9)
Statins, *n* (%)	46 (66.7)	13 (56.5)	15 (65.2)	18 (78.3)

Data are presented as mean ± standard deviation for normally distributed variables, and *n* (%) for categorical variables.

*BMI* Body Mass Index; *C-HIIT* Combined High-Intensity Interval Training; *C-MICT* Combined Moderate Intensity Continuous Training; *CON* Waitlist Control; *FPG* Fasting Plasma Glucose; *FBI* Fasting Blood Insulin; *HbA*_*1c*_ glycated haemoglobin; $\dot{V}$*O*_*2peak*_ peak oxygen consumption

### Intervention attendance and adherence

The number of sessions attended and the adherence to the prescribed intensity was high in both exercise groups during phase one: 96.7 ± 6.0% attendance to C-HIIT sessions and 82.0 ± 17.4% adherence to the intensity; 94.1 ± 6.2% attendance to C-MICT sessions and 87.0 ± 9.1% adherence to the intensity. However, both groups reduced their exercise participation during the self-directed phase (phase two), with a greater reduction in C-HIIT: C-HIIT 40.6 ± 25.6% attendance and 67.1 ± 34.6% adherence to the intensity; C-MICT 60.6 ± 27.5% attendance and 79.8 ± 20.9% adherence to the intensity. The most reported barriers to self-directed exercise, by both intervention groups, were lack of access to specialised equipment and competing time demands. On average, the participants attended 58% of the optional, once monthly supervised exercise training sessions at the University during phase two (C-HIIT 57%, C-MICT 62%).

### Phase one–8-weeks of supervised training

Table 2 shows the changes in vascular health from baseline to 8-weeks, between C-HIIT, C-MICT and CON. There were no significant between-group differences in the changes in haemodynamic indices, cfPWV, or ARP. A total of 41 participants were included in the FMD analysis (C-HIIT *n* = 11, C-MICT *n* = 15, CON *n* = 15); the remaining 28 participants were excluded due to poor image quality at baseline or inability to analyse using the automatic edge-detection software. Compared with the change in CON, C-HIIT significantly improved absolute and relative FMD (mean difference [MD] 0.004 mm [95% CI 0.002, 0.006], *p* = 0.002; MD 0.8% [0.1, 1.4] *p* = 0.025, respectively), as well as time to peak diameter (MD –18.3 s [–35, –1.6], *p* = 0.033). The improvement in relative FMD following C-MICT was of the same magnitude as the improvement following C-HIIT, but was not significantly different from CON (MD 0.8% [–0.1, 1.7] *p* = 0.080). There were no differences in the changes in any brachial artery FMD indices between C-MICT and CON, or between C-HIIT and C-MICT. These findings were unchanged when including only those participants who attended and adhered to at least 70% of the prescribed intervention (Table S1). Sensitivity analyses excluding participants with changes to cardiac medications showed that compared with CON, C-MICT significantly improved absolute and relative FMD (Table S2).

**Table 2:** Vascular health outcomes at baseline and after eight weeks of supervised exercise training or waitlist control (phase one)

	**C-HIIT**			**C-MICT**			**CON**			**Mean Difference**^a^**(95% CI) [sample sizes for comparator groups]**		
**Variable**	**Baseline**	**8-weeks**	**∆**	**Baseline**	**8-weeks**	**∆**	**Baseline**	**8-weeks**	**∆**	**C-HIIT—CON**	**C-MICT—CON**	**C-HIIT—C-MICT**
**Haemodynamic Indices**												
Heart rate (bpm)	63 ± 7	62 ± 8	–1 ± 5	62 ± 10	60 ± 9	–2 ± 4	67 ± 13	65 ± 13	–2 ± 4	0.7 (–1.7, 3.2) [23, 23]	0.4 (–1.8, 2.5) [23, 23]	0.4 (–2.0, 2.9) [23, 23]
bSBP (mmHg)	133 ± 12	133 ± 10	0 ± 9	131 ± 14	129 ± 13	–2 ± 8	133 ± 17	131 ± 14	–2 ± 14	1.8 (–3.9, 7.4) [23, 23]	–0.7 (–6.4, 5.0) [23, 23]	2.3 (–2.2, 6.7) [23, 23]
bDBP (mmHg)	77 ± 6	76 ± 7	–2 ± 5	77 ± 10	75 ± 9	–3 ± 6	76 ± 9	74 ± 8	–2 ± 9	1.1 (–2.8, 5.0) [23, 23]	0.1 (–3.9, 4.0) [23, 23]	1.0 (–2.1, 4.1) [23, 23]
MAP (mmHg)	96 ± 7	95 ± 7	–1 ± 6	95 ± 11	93 ± 9	–2 ± 6	94 ± 10	92 ± 8	–2 ± 11	1.8 (–2.2, 5.9) [23, 23]	–0.1 (–4.1, 4.2) [23, 23]	1.9 (–2.2, 5.9) [23, 23]
cSBP (mmHg)	120 ± 10	120 ± 9	0 ± 8	120 ± 12	118 ± 11	–2 ± 8	120 ± 16	119 ± 12	–1 ± 12	0.7 (–4.2, 5.6) [23, 23]	–1.2 (–6.2, 3.7) [23, 23]	1.7 (–2.7, 6.0) [23, 23]
cDBP (mmHg)	78 ± 6	77 ± 7	–1 ± 5	78 ± 10	76 ± 9	–3 ± 6	77 ± 9	75 ± 8	–2 ± 9	1.2 (–2.8, 5.1) [23, 23]	–0.1 (–4.0, 3.9) [23, 23]	1.2 (–1.9, 4.3) [23, 23]
cPP (mmHg)	42 ± 9	43 ± 7	1 ± 6	41 ± 8	42 ± 9	1 ± 4	43 ± 12	44 ± 11	1 ± 6	–0.2 (–3.1, 2.7) [23, 23]	–0.6 (–3.4, 2.2) [23, 23]	0.6 (–2.4, 3.2) [23, 23]
AIx (%)	26 ± 17	26 ± 7	0 ± 5	27 ± 7	26 ± 8	0 ± 5	25 ± 10	27 ± 11	2 ± 7	–1.9 (–5.6, 1.8) [21, 23]	–2.2 (–5.9, 1.4) [23, 23]	0.2 (–2.5, 3.0) [21, 23]
AIx@75 (%)	21 ± 5	20 ± 8	0 ± 5	20 ± 5	19 ± 7	–1 ± 4	20 ± 10	21 ± 12	1 ± 7	–1.2 (–5.1, 2.7) [21, 23]	–2.0 (–5.6, 1.6) [23, 23]	0.8 (–2.2, 3.8) [21, 23]
Forward pressure wave (mmHg)	28 ± 5	29 ± 6	1 ± 4	26 ± 5	26 ± 6	0 ± 3	30 ± 6	29 ± 5	–1 ± 4	1.9 (–0.4, 4.1) [23, 23]	0.2 (–1.8, 2.1) [23, 23]	1.7 (–0.4, 3.9) [23, 23]
Reflected pressure wave (mmHg)	18 ± 3	18 ± 3	1 ± 2	18 ± 3	18 ± 4	0 ± 2	18 ± 6	19 ± 5	0 ± 3	–0.0 (–1.5, 1.5) [23, 23]	–0.3 (–1.7, 1.1) [23, 23]	0.3 (–0.9, 1.5) [23, 23]
Reflection magnitude (%)	63 ± 9	62 ± 9	–1 ± 7	70 ± 13	71 ± 15	–1 ± 6	62 ± 13	66 ± 14	–4 ± 11	– 4.5 (–9.6, 0.6) [23, 23]	–2.1 (–7.6, 3.3) [23, 23]	– 2.6 (–6.5, 1.5) [23, 23]
**Arterial Stiffness**												
cfPWV (m·s^–1^)	8.7 ± 1.6	9.1 ± 1.3	0.4 ± 1.2	9.2 ± 1.3	9.2 ± 1.3	0.0 ± 0.6	9.6 ± 1.8	9.6 ± 1.6	–0.0 ± 1.2	–0.1 (–0.6, 0.7) [21, 21]	–0.0 (–0.6, 0.5) [21, 23]	0.2 (–0.3, 0.7) [21, 23]
**Aortic Reservoir Pressure**												
ARP (mmHg)	115 ± 10	114 ± 9	0 ± 9	114 ± 12	112 ± 11	–3 ± 8	114 ± 16	112 ± 11	–2 ± 13	1.7 (–3.2, 6.6) [23, 23]	– 0.9 (–5.8, 4.0) [23, 23]	2.5 (–1.9, 6.8) [23, 23]
ARP less DBP (mmHg)	34 ± 7	35 ± 6	1 ± 6	33 ± 6	33 ± 8	0 ± 3	34 ± 10	34 ± 7	0 ± 6	1.0 (–1.8, 3.8) [23, 23]	–0.7 (–3.3, 2.0) [23, 23]	1.5 (–1.2, 4.3) [23, 23]
ARP AUC (mmHg)	10.6 ± 2.3	10.9 ± 2.4	0.3 ± 2.2	9.8 ± 2.3	9.5 ± 2.5	–0.3 ± 1.4	10.3 ± 3.2	10.0 ± 2.4	–0.3 ± 2.4	0.7 (–0.4, 1.8) [23, 23]	–0.2 (–1.2, 0.8) [23, 23]	0.8 (–0.3, 1.9) [23, 23]
**Flow-Mediated Dilation**												
Resting diameter (mm)	4.1 ± 0.7	4.2 ± 0.7	0.2 ± 0.4	4.4 ± 1.1	4.4 ± 1.1	0 ± 0.3	4.6 ± 0.5	4.7 ± 0.8	0.1 ± 0.7	0.0 (–0.5, 0.6) [11, 15]	–0.1 (–0.5, 0.3) [15, 15]	0.2 (–0.1, 0.4) [11, 15]
FMD (mm)	0.1 ± 0.1	0.2 ± 0.1	0.03 ± 0.03	0.2 ± 0.1	0.2 ± 0.1	0.03 ± 0.1	0.2 ± 0.1	0.2 ± 0.1	–0.01 ± 0.1	0.04* (0.02, 0.06) [11, 15]	0.04* (0.00, 0.07) [15, 15]	0.00 (–0.04, 0.04) [11, 15]
FMD (%)	3.6 ± 1.7	4.2 ± 1.4	0.6 ± 0.9	3.9 ± 2.0	4.4 ± 2.5	0.6 ± 1.4	3.8 ± 2.1	3.5 ± 1.8	–0.3 ± 0.8	0.8* (0.1, 1.4) [11, 15]	0.8 (–0.1, 1.7) [15, 15]	–0.1 (–1.1, 1.0) [11, 15]
Resting blood flow (ml·s^−1^)	1.2 ± 0.9	1.2 ± 0.7	0 ± 1.1	0.9 ± 0.5	1.3 ± 0.9	0.3 ± 0.8	1.1 ± 0.6	1.4 ± 0.9	0.4 ± 0.7	–0.3 (–0.9, 0.3) [11, 15]	–0.0 (–0.6, 0.5) [15, 15]	–0.2 (–0.9, 0.5) [11, 15]
Peak blood flow (ml·s^−1^)	3.9 ± 1.8	5.4 ± 2.2	1.4 ± 2.5	4.6 ± 2.6	4.9 ± 3.8	0.6 ± 3.0	5.0 ± 2.9	5.9 ± 2.4	0.9 ± 2.9	0.1 (–2.0, 1.8) [11, 15]	–0.5 (–2.6, 1.7) [15, 15]	0.7 (–1.7, 3.1) [11, 15]
FMD SR_AUC_ (10^3^·s^−1^)	16.5 ± 8.4	14.8 ± 6.0	–1.7 ± 6.1	12.1 ± 6.0	11.3 ± 6.8	–0.6 ± 7.2	13.4 ± 5.7	16.2 ± 8.3	2.9 ± 6.6	–3.5 (–8.7, 1.6) [11, 15]	–3.8 (–9.0, 1.5) [15, 15]	1.0 (–4.2, 6.1) [11, 15]
Time to peak diameter (s)	60 ± 30	41 ± 13	–20 ± 28	47 ± 21	46 ± 24	–2 ± 14	56 ± 24	56 ± 28	1 ± 22	–18.3 (–35.0, –1.6) [11, 15]	–3.9 (–18.0, 10.2) [15, 15]	–11.5 (–25.5, 2.5) [11, 15]
**Glycaemic Control, Body Mass & Composition, & Cardiorespiratory Fitness**												
HbA_1c_ (%)	8.6 ± 2.2	8.4 ± 2.0	–0.2 ± 1.2	9.0 ± 1.8	8.3 ± 1.9	–0.7 ± 1.3	8.1 ± 1.3	8.8 ± 1.5	0.7 ± 0.8	–0.7 (–1.3, –0.2) [22,23]*	–1.2 (–1.9, –0.6) [22,23]*	0.4 (–0.3, 1.2) [22, 22]
HbA_1c_ (mmol·mol)	70 ± 24	69 ± 21	–2 ± 13	75 ± 20	67 ± 21	–8 ± 14	65 ± 14	72 ± 16	8 ± 9	–8 (–15, –2) [22, 23]*	–14 (–21, –6) [22,23]*	5 (–3, 13) [22, 22]
FPG (mmol·L)	9.0 ± 2.9	8.9 ± 2.8	0.0 ± 2.3	9.1 ± 2.8	8.5 ± 2.3	–0.7 ± 2.9	8.6 ± 2.6	8.8 ± 2.9	0.3 ± 2.8	–0.1 (–1.5, 1.3) **[22,22]**	–0.6 (–2.1, 0.8) **[23,22]**	0.6 (–0.8, 1.9) **[22,23]**
Body mass (kg)	93.2 ± 15.9	93.8 ± 14.8	0.5 ± 2.5	97.5 ± 19.6	97.4 ± 19.4	–0.1 ± 1.4	103.0 ± 21.6	103.4 ± 22.2	0.4 ± 2.6	–0.0 (–1.6, 1.6) [23, 23]	–0.4 (–1.7, 0.8) [23, 23]	0.4 (–0.7, 1.6) [23, 23]
Body fat (%)	39.3 ± 6.3	38.5 ± 6.4	–0.9 ± 1.2	39.2 ± 8.4	38.8 ± 8.4	–0.4 ± 1.0	40.3 ± 6.9	41.0 ± 6.9	0.7 ± 1.4	**–1.6 (–2.4, –0.8) **[23, 23]*	**–1.1 (–1.8, –0.4) **[23, 23]*	–0.5 (–1.2, 0.2) [23, 23]
$\dot{{\text{V}}}$O_2peak_ (mL·kg^–1^·min^–1^)	24.3 ± 5.1	24.5 ± 4.3	0.2 ± 3.8	24.7 ± 7.0	25.5 ± 7.3	0.8 ± 1.9	23.3 ± 5.3	22.2 ± 4.9	–1.0 ± 1.4	**1.5 (0.0, 3.0) **[23, 23]*	**1.9 (0.9, 2.9) **[23, 23]*	–0.6 (–2.4, 1.1) [23, 23]

Data are presented as mean ± standard deviation. *Boldface indicates statistical significance (*p* ≤ 0.05)

^a^Mean Difference calculated as difference between change scores for C-HIIT and CON, C-MICT and CON, and C-HIIT and C-MICT after 8-weeks, respectively

***∆*** change score; *AIx* augmentation index; *AIx@75* augmentation index adjusted for a heart rate of 75 bpm; *ARP* aortic reservoir pressure; *AUC* area under the curve; *bDBP* brachial diastolic blood pressure; *bSBP* brachial systolic blood pressure; *cDBP* central diastolic blood pressure; *cfPWV* carotid-femoral pulse wave velocity; *C-HIIT* Combined High-Intensity Interval Training; *C-MICT* Combined Moderate Intensity Continuous Training; *CON* Waitlist Control; *cPP* central pulse pressure; *cSBP* central systolic blood pressure; *FPG* fasting plasma glucose; *FMD* flow-mediated dilation; *HbA*_*1c*_ glycated haemoglobin; *MAP* mean arterial pressure; *SR*_*AUC*_ shear rate area under the curve; $\dot{V}$*O*_*2peak*_ peak oxygen consumption

### Phase two—8-weeks of supervised training and 10-months of self-directed training

Table 3 shows the changes in vascular health from baseline to 12-months, between C-HIIT and C-MICT. After 12-months of exercise training, there was a significant reduction in brachial and cDBP (MD –2.3 mmHg [–4.2, –0.5], *p* = 0.014; MD –2.4 mmHg [–4.3, –0.5], *p* = 0.013, respectively), MAP (MD –2.3 mmHg [–4.5, –0.03], *p* = 0.047), and ARP (MD –5.5 mmHg [–8.1, –2.9], *p* < 0.001) in C-HIIT and C-MICT, with no difference between groups. There was also a significant increase in the forward pressure wave (MD 1.5 mmHg [0.1, 2.9], *p* = 0.042) and reduction in reflection magnitude (MD –4.8% [–8.4, –1.2], *p* = 0.009) in C-HIIT and C-MICT, with no difference between groups. There was a significant group × time effect for the change in cfPWV over 12-months favouring C-MICT (*p* = 0.018); however, this was driven by an increase of 0.6 m·s^–1^ in the C-HIIT group rather than a reduction in C-MICT (﻿–0.2 m·s^–1^). There were no differences in the changes in any brachial artery FMD indices over time or between groups. In participants who attended and adhered to at least 50% of the prescribed intervention in both phases, the magnitude of improvement in vascular health after 12-months of exercise training was greater than in the main analyses but did not reach statistical significance (Table S3). Sensitivity analyses excluding participants with changes to cardiac medications demonstrated similar findings to the main analyses (Table S4).

**Table 3:** Vascular health outcomes at baseline and after 12-months (8-weeks supervised training and 10-months self-directed training), including waitlist control participants re-randomised (phase two)

	**C-HIIT**			**C-MICT**			**Mean Time Difference**^**a**^** (95% CI) [sample sizes for comparator groups]**	***p*-value, Time**	***p*-value, Group × Time**
	**Baseline**	**12 months**	**∆**	**Baseline**	**12 months**	**∆**			
**Haemodynamic Indices**									
Heart rate (bpm)	63 ± 9	63 ± 9	0 ± 5	62 ± 9	62 ± 10	0 ± 6	–0.3 (–1.8, 1.2) [29, 33]	0.692	0.938
bSBP (mmHg)	132 ± 13	131 ± 15	–1 ± 12	130 ± 13	126 ± 15	–4 ± 13	–2.3 (–5.6, 0.9) [29, 33]	0.148	0.410
bDBP (mmHg)	76 ± 8	74 ± 9	–2 ± 7	77 ± 8	74 ± 9	–3 ± 7	–2.3* (–4.2, –0.5) [29, 33]	0.014*	0.575
MAP (mmHg)	95 ± 9	93 ± 9	–2 ± 8	95 ± 9	92 ± 10	–3 ± 9	–2.3* (–4.5, –0.03) [29, 33]	0.047*	0.493
cSBP (mmHg)	120 ± 12	120 ± 13	–1 ± 11	119 ± 12	115 ± 14	–4 ± 12	–2.1 (–5.0, 0.8) [29, 33]	0.152	0.337
cDBP (mmHg)	77 ± 8	75 ± 9	–2 ± 7	78 ± 8	75 ± 9	–3 ± 8	–2.4* (–4.3, –0.5) [29, 33]	0.013*	0.675
cPP (mmHg)	44 ± 9	45 ± 10	–1 ± 6	41 ± 9	40 ± 10	–1 ± 7	–0.2 (–1.5, 1.9) [29, 33]	0.821	0.229
AIx (%)	28 ± 9	30 ± 9	2 ± 8	25 ± 8	24 ± 10	–1 ± 8	0.5 (–1.6, 2.6) [29, 31]	0.626	0.121
AIx@75 (%)	22 ± 9	24 ± 10	2 ± 9	19 ± 9	18 ± 10	–1 ± 10	0.5 (–1.9, 2.9) [29, 31]	0.688	0.134
Forward pressure wave (mmHg)	28 ± 5	30 ± 6	2 ± 5	26 ± 5	27 ± 6	1 ± 6	1.5* (0.1, 2.9) [29, 32]	0.042*	0.308
Reflected pressure wave (mmHg)	18 ± 3	18 ± 4	0 ± 4	17 ± 3	17 ± 4	–1 ± 4	–0.4 (–1.2, 0.6) [29, 32]	0.420	0.228
Reflection magnitude (%)	64 ± 5	60 ± 4	–5 ± 6	68 ± 5	63 ± 8	–5 ± 7	–4.8* (–8.4, –1.2) [32, 61]	0.009*	0.850
**Arterial Stiffness**									
cfPWV (m·s^–1^)	9.2 ± 1.6	9.8 ± 1.8	0.6 ± 1.3	9.3 ± 1.5	9.1 ± 1.8	–0.2 ± 1.4	0.2 (–0.2, 0.6) [29, 30]	0.270	**0.018**^**#**^
**Aortic Reservoir Pressure**									
ARP (mmHg)	114 ± 11	110 ± 12	–4 ± 10	113 ± 11	106 ± 12	–7 ± 10	–5.5* (–8.1, –2.9) [29, 30]	< 0.001*	0.147
ARP less DBP (mmHg)	35 ± 7	35 ± 8	1 ± 6	33 ± 7	31 ± 8	–1 ± 6	–0.3 (–1.8, 1.3) [29, 33]	0.712	0.157
ARP AUC (mmHg)	10.5 ± 2.4	10.7 ± 2.6	0.2 ± 1.9	9.6 ± 2.4	9.0 ± 2.7	–0.6 ± 2.1	–0.2 (–0.7, 0.3) [29, 33]	0.439	0.113
**Flow-Mediated Dilation**									
Resting diameter (mm)	4.4 ± 1.0	4.3 ± 1.0	0.1 ± 0.6	4.5 ± 1.0	4.4 ± 1.1	0 ± 0.7	–0.1 (–0.2, 0.3) [19, 20]	0.617	0.805
FMD (mm)	0.1 ± 0.1	0.2 ± 0.1	0.01 ± 0.07	0.2 ± 0.1	0.2 ± 0.1	0.01 ± 0.06	0.01 (–0.02, 0.04) [19, 20]	0.421	0.958
FMD (%)	3.5 ± 1.9	3.8 ± 2.0	0.2 ± 2.3	3.7 ± 2.2	4.4 ± 2.6	0.6 ± 2.7	0.4 (–0.5, 1.3) [19, 20]	0.372	0.650
Resting blood flow (ml·s^−1^)	1.5 ± 1.3	1.4 ± 1.5	–0.1 ± 1.9	1.0 ± 1.3	2.0 ± 1.9	1.0 ± 2.2	0.5 (–0.2, 1.1) [19, 20]	0.549	0.782
Peak blood flow (ml·s^−1^)	4.8 ± 2.4	5.1 ± 2.8	0.3 ± 2.9	4.4 ± 2.4	5.6 ± 3.3	1.2 ± 3.4	0.7 (–0.3, 1.8) [19, 20]	0.148	0.374
FMD SR_AUC_ (10^3^·s^−1^)	15.8 ± 6.8	18.7 ± 7.3	2.9 ± 4.9	11.6 ± 6.8	11.6 ± 8.0	0 ± 5.9	1.4 (–0.4, 3.2) [19, 20]	0.111	0.108
Time to peak diameter (s)	56 ± 24	65 ± 28	9 ± 32	49 ± 24	39 ± 37	–10 ± 37	–0.7 (–12.0, 10.6) [19, 20]	0.900	0.105
**Glycaemic Control, Body Mass & Composition, & Cardiorespiratory Fitness**									
HbA_1c_ (%)	8.8 ± 2.1	9.0 ± 2.2	0.1 ± 1.8	8.8 ± 1.6	8.4 ± 1.9	–0.5 ± 1.9	–0.2 (–0.7, 0.3) [28, 32]	0.412	0.189
HbA_1c_ (mmol·mol)	73 ± 23	75 ± 24	1 ± 19	73 ± 18	68 ± 21	–6 ± 21	–2 (–7, 3) [28, 32]	0.412	0.189
FPG (mmol·L)	9.1 ± 3.0	9.3 ± 2.7	0.1 ± 3.3	9.1 ± 2.8	8.7 ± 1.6	–0.5 ± 3.5	–0.2 (–1.1, 0.6) [29, 33]	0.606	0.459
Body mass (kg)	97.0 ± 17.9	95.8 ± 15.6	–1.0 ± 4.3	99.6 ± 22.1	94.3 ± 19.9	–2.0 ± 4.6	–1.5 (–2.6, –0.3) [29, 33]	**0.013**	0.390
Body fat (%)	40.2 ± 6.7	40.3 ± 7.0	0.0 ± 2.0	39.1 ± 8.0	38.0 ± 7.6	0.0 ± 2.1	0.0 (–0.5, 0.5) [29, 33]	0.988	0.914
$\dot{{\text{V}}}$O_2peak_ (mL·kg^–1^·min^–1^)	22.6 ± 4.7	22.5 ± 4.6	–0.1 ± 3.4	24.6 ± 6.5	25.4 ± 6.4	0.3 ± 3.6	0.1 (–0.8, 1.0) [29, 33]	0.824	0.687

Data are presented as mean ± standard deviation. *Boldface indicates statistical significance (*p* ≤ 0.05). ^#^C-MICT significantly different from C-HIIT (*p* ≤ 0.05)

^a^Mean Time Difference calculated as 12 months minus baseline (pooled effects of exercise)

***∆*** change score; *AIx* augmentation index; *AIx@75* augmentation index adjusted for a heart rate of 75 bpm; *ARP* aortic reservoir pressure; *AUC* area under the curve; *bDBP* brachial diastolic blood pressure; *bSBP* brachial systolic blood pressure; *cDBP* central diastolic blood pressure; *cfPWV* carotid-femoral pulse wave velocity; *C-HIIT* Combined High-Intensity Interval Training; *C-MICT* Combined Moderate Intensity Continuous Training; *CON* Waitlist Control; *cPP* central pulse pressure; *cSBP* central systolic blood pressure; *FPG* fasting plasma glucose; *FMD* flow-mediated dilation; *HbA*_*1c*_ glycated haemoglobin; *MAP* mean arterial pressure; *SR*_*AUC*_ shear rate area under the curve; $\dot{V}$*O*_*2peak*_ peak oxygen consumption

## Discussion

This is the first trial to investigate the short- and long-term effects of low-volume, combined aerobic and resistance high-intensity interval training (C-HIIT) on markers of vascular health in people with T2D. Effects were compared with combined moderate intensity continuous training (C-MICT) and a waitlist control (CON). In the short-term (8-weeks), supervised C-HIIT and C-MICT both increased brachial artery FMD compared with CON. There were no significant differences between C-HIIT and C-MICT when these groups were directly compared. Despite the reported benefits of supervised exercise for vascular health in people with T2D (Way 2020; Ghardashi Afousi et al. 2018), there were no other changes after 8-weeks. In the long-term (12-months; 8-weeks supervised, 10-months self-directed), there were comparable improvements in haemodynamic indices and ARP with both C-HIIT and C-MICT. However, only C-MICT induced a reduction in arterial stiffness after 12-months, as measured by cfPWV. These data suggest some improvements to vascular health result from combined aerobic and resistance exercise interventions in people with T2D, and that exercise performed at either a moderate- or high-intensity can be beneficial.

We observed an improvement in vascular function after 8-weeks of C-HIIT compared with CON (~ 0.6% increase in FMD); a 1% increase in vascular function is associated with a clinically meaningful reduction in the risk of cardiovascular events (Inaba et al. 2010). We also observed an improvement in arterial stiffness after 12-months of C-MICT compared with C-HIIT. However, this was driven by an increase in C-HIIT, rather than a reduction in C-MICT. The 6.5% relative increase observed following 12-months of C-HIIT (from 9.2 to 9.8 m·s^–1^) is in line with the increases expected with normal ageing (Díaz et al. 2014), so while the *change* in C-MICT was not clinically meaningful (from 9.3 to 9.1 m·s^–1^)(Vlachopoulos et al. 2014), the *maintenance* of arterial stiffness in this group is important. Despite employing the same *vascular* methodology, the changes we observed in vascular function and arterial stiffness are in line with some (Way et al. 2016; Magalhaes et al. 2019; Barone Gibbs et al. 2012), but not all studies (Way et al. 2020; Sawyer et al. 1985; Mitranun et al. 2014). However, there is a large degree of heterogeneity in the *exercise* protocols used in the literature, with the majority of studies investigating the effect of exercise intensity on vascular health focussing on aerobic-only exercise. These studies have primarily reported significant increases in vascular function following short-term HIIT, compared with MICT (Ghardashi Afousi et al. 2018; Sawyer, et al. 1985; Mitranun et al. 2014), and comparable reductions in arterial stiffness with both HIIT and MICT (Way 2020). Additionally, the measurement site of the vascular indices may have impacted the changes observed. Specifically, the magnitude of the effect of exercise on arterial stiffness is greater peripherally than centrally (Ashor et al. 2015), due to the greater shear stress-enhanced release of nitric oxide in the peripheral exercising limbs and the nitric oxide-producing small conduit arteries (Green et al. 2004). Given the greater predictive strength of central versus peripheral vascular indices for future cardiovascular risk, the present study used central measures and thus would not have been able to detect changes in the peripheral vasculature as a result of the interventions.

We did not find a superior effect of HIIT compared with MICT on vascular health. The combination of aerobic and resistance training in the present trial may have dampened the effects of exercise on vascular function (Casey et al. 2007; Rakobowchuk, et al. 1985) and arterial stiffness (Miyachi 2013). Importantly, combining resistance and aerobic training did not negatively impact vascular health, and resistance training in this population provides significant benefits for glycaemic control and skeletal muscle mass (Hordern et al. 2012). There are studies that have successfully utilised the two modalities concurrently for vascular benefit (Maiorana et al. 2001; Okada et al. 2010; Francois et al. 2018). It appears that prescribing aerobic and resistance training on different days may be of benefit for vascular health. For example, Francois et al. (2018) found a significant decrease in cfPWV after 12-weeks of low-volume aerobic HIIT combined with resistance training (completed on separate days) in people with T2D (Francois et al. 2018). In the present study, C-MICT completed two sessions of aerobic-only training, in addition to two sessions of combined aerobic and resistance training. This may have contributed to the reduction in cfPWV observed after 12-months in this group, while C-HIIT, whose sessions involved only combined training, showed no change in cfPWV. When examining other studies that have seen improvements in vascular health with combined aerobic and resistance training, participants in the study by Maiorana et al. (2001) had a significantly lower FMD% at baseline compared with the participants in the current trial (1.7 ± 0.5% versus 3.8 ± 1.9%), therefore, allowing more opportunity for change (Maiorana et al. 2001). The participants in the study by Okada et al. (2010) exercised for up to 375 min per week for 3-months, providing a much greater exercise stimulus than the current trial (Okada et al. 2010).

After 12-months, there were similar decreases in haemodynamic indices for both C-HIIT and C-MICT. Specifically, we observed reductions in both peripheral and central diastolic blood pressure by  ~ 2 mmHg, with similar decreases in systolic blood pressure (though these were not statistically significant). A reduction in diastolic blood pressure of  ≥ 0.9 mmHg has been shown to be clinically meaningful, reducing the frequency of major cardiovascular events by 10% in people with T2D (Turnbull et al. 2005). Overall, these positive changes also led to a reduction in MAP. Given the association between increased MAP and cardiovascular event risk in adults with T2D (Kodama et al. 2014), a reduction in haemodynamic indices in both exercise groups over 12-months suggests an improvement in traditional risk factor control in this sample.

Aortic reservoir pressure (ARP) independently predicts adverse cardiovascular events in people with cardiovascular disease (Hametner et al. 2014), yet few studies have investigated the impact of exercise training on ARP (Ramos et al. 2016). There were no changes in ARP after 8-weeks, but there were improvements (reductions) after 12-months following both C-HIIT and C-MICT. Mechanistically, this change may have been mediated by reductions in aortic stiffness (cfPWV), though there was an increase of  ~ 0.6 m·s^–1^ from baseline in the C-HIIT group at 12-months. Alternatively, exercise training may have decreased systemic (peripheral) vascular resistance, with both aerobic and resistance training previously shown to reduce systemic vascular resistance because of decreased autonomic nervous system activity (Fagard 2006).

Our trial has some limitations. The 8-week duration for the supervised exercise phase was based on assessing potential changes in glycaemic control (as the main aim of the E4D Trial), but this may not have been long enough to induce significant improvements in haemodynamic indices and arterial stiffness (Ramos et al. 2015). Additionally, as these data are secondary outcomes, the study was not powered for these outcomes and a larger sample size may be needed to detect changes in vascular health. Furthermore, as the aim of the trial was to compare the effect of a low-volume HIIT protocol, with a higher dose of MICT as per the current exercise guidelines (Hordern et al. 2012), the groups were not energy matched and, therefore, had different external loads. This is likely to have influenced the outcomes in this study. There was also poor adherence in C-HIIT during the self-directed phase (phase two), with participants completing less than 40% of the prescribed sessions. Given this equates to 1.2 sessions and 31.2 min of exercise per week, this is likely to be insufficient to elicit benefits for vascular function and arterial stiffness. However, we did observe improvements to traditional risk factor control (e.g., brachial and central blood pressures). Long-term exercise participation is crucial to prevent early deterioration in vascular health and subsequent elevation in cardiovascular disease risk (Shinji et al. 2007). In agreement with this, participants who attended and adhered to more than 50% of the prescribed intervention over 12-months had greater improvements in vascular health than those with lower attendance/adherence, though these were not statistically significant due to small sample size. Therefore, strategies to improve adherence should be investigated so the effect of long-term C-HIIT on vascular health can be appropriately assessed, particularly in self-directed settings that are likely to be more real-world applicable. A further limitation is the lack of control group during phase two. However, this was done as it was considered ethical to provide all trial participants with access to the “intervention”, as well as to maintain participant engagement in the trial.

## Conclusion

The Exercise for Type 2 Diabetes (E4D) Trial is the first to investigate the short- and long-term effects of high-intensity interval and moderate-intensity continuous combined aerobic and resistance training on vascular health including haemodynamic indices, arterial stiffness, ARP, and brachial artery FMD. Only C-HIIT was superior to waitlist control for improvements in brachial artery FMD after 8-weeks, but the magnitude of improvement with C-MICT and C-HIIT did not differ. Contrary to the hypothesis and other research in the field, there were no other differences in changes in vascular health following 8-weeks of supervised C-HIIT or C-MICT, compared with control. After 12-months of exercise training, both C-HIIT and C-MICT demonstrated improvements in haemodynamic indices, with C-MICT superior to C-HIIT for reductions in arterial stiffness. These results indicate that both interventions are beneficial for improving haemodynamic indices, but not vascular function, in individuals with T2D after 12-months.

### Supplementary Information

Below is the link to the electronic supplementary material.

Supplementary file1 (DOCX 109 KB)

## Data Availability

The data that support the findings of this study are available from the corresponding author upon reasonable request.

## Abbreviations

AEP: Accredited exercise physiologist
AIx: Augmentation index
AIx@75: Augmentation index adjusted for heart rate of 75 beats per minute
ANCOVA: Analysis of covariance
ARP: Aortic reservoir pressure
bDBP: Brachial diastolic blood pressure
bSBP: Brachial systolic blood pressure
C-HIIT: Combined aerobic and resistance high-intensity interval training
C-MICT: Combined aerobic and resistance moderate intensity continuous training
cDBP: Central diastolic blood pressure
cfPWV: Carotid-femoral pulse wave velocity
cPP: Pulse pressure
cSBP: Central systolic blood pressure
CON: Waitlist control
CPET: Cardiopulmonary exercise test
E4D: Exercise for Type 2 Diabetes
FMD: Flow-mediated dilation
HbA_1c_: Glycated haemoglobin
HIIT: High-intensity interval training
HR: Heart rate
HR_peak_: Heart rate peak
MAP: Mean arterial pressure
MD: Mean difference
MICT: Moderate intensity continuous training
Pb: Reflected pressure wave
Pf: Forward pressure wave
PWA: Pulse wave analysis
RPE: Rating of perceived exertion
SR_AUC_: Shear rate area under the curve
T2D: Type 2 diabetes
$\dot{{\text{V}}}$O_2peak_: Peak oxygen uptake

## References

[CR1] Armijo-Olivo S, Warren S, Magee D (2009) Intention to treat analysis, compliance, drop-outs and how to deal with missing data in clinical research: a review. Phys Ther Rev 14(1):36–49 10.1179/174328809X405928

[CR2] Ashor AW et al (2015) Exercise modalities and endothelial function: a systematic review and dose-response meta-analysis of randomized controlled trials. Sports Med 45(2):279–296 25281334 10.1007/s40279-014-0272-9

[CR3] Barone Gibbs B et al (2012) A randomized trial of exercise for blood pressure reduction in type 2 diabetes: effect on flow-mediated dilation and circulating biomarkers of endothelial function. Atherosclerosis 224(2):446–453 22889573 10.1016/j.atherosclerosis.2012.07.035 PMC3459298

[CR4] Black MA et al (2008) importance of measuring the time course of flow-mediated dilatation in humans. Hypertension 51(2):203–210 18086954 10.1161/HYPERTENSIONAHA.107.101014

[CR5] Casey DP et al (2007) Effect of resistance training on arterial wave reflection and brachial artery reactivity in normotensive postmenopausal women. Eur J Appl Physiol 100(4):403–408 17394009 10.1007/s00421-007-0447-2

[CR6] Cox ER et al (2022) Effects of fitness and fatness on age-related arterial stiffening in people with type 2 diabetes. Clinical Obesity 12(3):e12519 35293141 10.1111/cob.12519 PMC9285462

[CR7] de Mello MB et al (2021) Effect of high-intensity interval training protocols on VO(2)max and HbA1c level in people with type 2 diabetes: a systematic review and meta-analysis. Ann Phys Rehabil Med 65(5):101586 34648979 10.1016/j.rehab.2021.101586

[CR8] Díaz A et al (2014) Reference values of pulse wave velocity in healthy people from an urban and rural argentinean population. Int J Hypertens 2014:653239 25215227 10.1155/2014/653239 PMC4158305

[CR9] Fagard RH (2006) Exercise is good for your blood pressure: effects of endurance training and resistance training. Clin Exp Pharmacol Physiol 33(9):853–856 16922820 10.1111/j.1440-1681.2006.04453.x

[CR10] Francois ME et al (2018) Cardiovascular benefits of combined interval training and post-exercise nutrition in type 2 diabetes. J Diabetes Complications 32(2):226–233 29198993 10.1016/j.jdiacomp.2017.10.002

[CR11] Ghardashi Afousi A et al (2018) Improved brachial artery shear patterns and increased flow-mediated dilatation after low-volume high-intensity interval training in type 2 diabetes. Exp Physiol 103(9):1264–1276 29932275 10.1113/EP087005

[CR12] Gibala MJ, McGee SL (2008) Metabolic adaptations to short-term high-intensity interval training: a little pain for a lot of gain? Exerc Sport Sci Rev 36(2):58–63 18362686 10.1097/JES.0b013e318168ec1f

[CR13] Green DJ et al (2004) Effect of exercise training on endothelium-derived nitric oxide function in humans. J Physiol 561(Pt 1):1–25 15375191 10.1113/jphysiol.2004.068197 PMC1665322

[CR14] Hadi HA, Suwaidi JA (2007) Endothelial dysfunction in diabetes mellitus. Vasc Health Risk Manag 3(6):853–876 18200806 PMC2350146

[CR15] Hametner B et al (2014) Reservoir and excess pressures predict cardiovascular events in high-risk patients. Int J Cardiol 171(1):31–36 24315153 10.1016/j.ijcard.2013.11.039

[CR16] Holder SM et al (2021) Reference intervals for brachial artery flow-mediated dilation and the relation with cardiovascular risk factors. Hypertension 77:1469–1480 33745297 10.1161/HYPERTENSIONAHA.120.15754

[CR17] Hordern MD et al (2012) Exercise prescription for patients with type 2 diabetes and pre-diabetes: a position statement from Exercise and Sport Science Australia. J Sci Med Sport 15(1):25–31 21621458 10.1016/j.jsams.2011.04.005

[CR18] Inaba Y, Chen JA, Bergmann SR (2010) Prediction of future cardiovascular outcomes by flow-mediated vasodilatation of brachial artery: a meta-analysis. Int J Cardiovasc Imaging 26(6):631–640 20339920 10.1007/s10554-010-9616-1

[CR19] Jang JE et al (2019) effectiveness of exercise intervention in reducing body weight and glycosylated hemoglobin levels in patients with type 2 diabetes mellitus in Korea: a systematic review and meta-analysis. Diabetes Metab J 43(3):302–318 30604592 10.4093/dmj.2018.0062 PMC6581545

[CR20] Kodama S et al (2014) Meta-analysis of the quantitative relation between pulse pressure and mean arterial pressure and cardiovascular risk in patients with diabetes mellitus. Am J Cardiol 113(6):1058–1065 24462067 10.1016/j.amjcard.2013.12.005

[CR21] Lenters-Westra E et al (2011) One in five laboratories using various hemoglobin a1c methods do not meet the criteria for optimal diabetes care management. Diabetes Technol Ther 13(4):429–433 21355726 10.1089/dia.2010.0148

[CR22] Magalhaes JP et al (2019) Effects of combined training with different intensities on vascular health in patients with type 2 diabetes: a 1-year randomized controlled trial. Cardiovasc Diabetol 18(1):34 30885194 10.1186/s12933-019-0840-2 PMC6423850

[CR23] Maiorana A et al (2001) The effect of combined aerobic and resistance exercise training on vascular function in type 2 diabetes. J Am Coll Cardiol 38(3):860–866 11527646 10.1016/S0735-1097(01)01439-5

[CR24] Mitranun W et al (2014) Continuous vs interval training on glycemic control and macro- and microvascular reactivity in type 2 diabetic patients. Scand J Med Sci Sports 24(2):e69-76 24102912 10.1111/sms.12112

[CR25] Miyachi M (2013) Effects of resistance training on arterial stiffness: a meta-analysis. Br J Sports Med 47(6):393–396 22267567 10.1136/bjsports-2012-090488

[CR26] Nolan RC et al (2016) Self-reported physical activity using the international physical activity questionnaire (IPAQ) in Australian adults with type 2 diabetes, with and without peripheral neuropathy. Can J Diabetes 40(6):576–579 27658764 10.1016/j.jcjd.2016.05.013

[CR27] Okada S et al (2010) Effect of exercise intervention on endothelial function and incidence of cardiovascular disease in patients with type 2 diabetes. J Atheroscler Thromb 17(8):828–833 20467191 10.5551/jat.3798

[CR28] Rakobowchuk, M., et al., *Endothelial function of young healthy males following whole body resistance training.* J Appl Physiol (1985), 2005. **98**(6): p. 2185–90. 10.1152/japplphysiol.01290.2004 15677730

[CR29] Ramos JS et al (2015) The impact of high-intensity interval training versus moderate-intensity continuous training on vascular function: a systematic review and meta-analysis. Sports Med 45(5):679–692 25771785 10.1007/s40279-015-0321-z

[CR30] Ramos JS et al (2016) 12 min/week of high-intensity interval training reduces aortic reservoir pressure in individuals with metabolic syndrome: a randomized trial. J Hypertens 34(10):1977–1987 27467767 10.1097/HJH.0000000000001034

[CR31] Sawyer BJ et al (1985) Effects of high-intensity interval training and moderate-intensity continuous training on endothelial function and cardiometabolic risk markers in obese adults. J Appl Physiol 121(1):279–288 10.1152/japplphysiol.00024.2016 PMC4967258 27255523

[CR32] Shinji S et al (2007) Adherence to a home-based exercise program and incidence of cardiovascular disease in type 2 diabetes patients. Int J Sports Med 28(10):877–879 17436204 10.1055/s-2007-964967

[CR33] The Reference Values for Arterial Stiffness Collaboration (2010) Determinants of pulse wave velocity in healthy people and in the presence of cardiovascular risk factors: ‘establishing normal and reference values.’ Eur Heart J 31(19):2338–2350 20530030 10.1093/eurheartj/ehq165 PMC2948201

[CR34] Thijssen DH et al (2009a) Brachial artery blood flow responses to different modalities of lower limb exercise. Med Sci Sports Exerc 41(5):1072–1079 19346980 10.1249/MSS.0b013e3181923957

[CR35] Thijssen DHJ et al (2009b) Does arterial shear explain the magnitude of flow-mediated dilation?: a comparison between young and older humans. Am J Physiol-Heart and Circ Physiol 296(1):H57–H64 19028795 10.1152/ajpheart.00980.2008 PMC2637778

[CR36] Thijssen DH et al (2011) Assessment of flow-mediated dilation in humans: a methodological and physiological guideline. Am J Physiol Heart Circ Physiol 300(1):H2-12 20952670 10.1152/ajpheart.00471.2010 PMC3023245

[CR37] Thomas N, Alder E, Leese GP (2004) Barriers to physical activity in patients with diabetes. Postgrad Med J 80(943):287 15138320 10.1136/pgmj.2003.010553 PMC1742997

[CR38] Townsend RR et al (2015) Recommendations for improving and standardizing vascular research on arterial stiffness: a scientific statement from the american heart association. Hypertension 66(3):698–722 26160955 10.1161/HYP.0000000000000033 PMC4587661

[CR39] Turnbull F et al (2005) Effects of different blood pressure-lowering regimens on major cardiovascular events in individuals with and without diabetes mellitus: results of prospectively designed overviews of randomized trials. Arch Intern Med 165(12):1410–1419 15983291 10.1001/archinte.165.12.1410

[CR40] Vlachopoulos C, Aznaouridis K, Stefanadis C (2014) Aortic stiffness for cardiovascular risk prediction: just measure it, just do it! J Am Coll Cardiol 63(7):647–649 24239659 10.1016/j.jacc.2013.10.040

[CR41] Way KL et al (2016) The effect of exercise on vascular function and stiffness in type 2 diabetes: a systematic review and meta-analysis. Curr Diabetes Rev 12(4):369–383 26279493 10.2174/1573399811666150817124601

[CR42] Way KL et al (2019) The effect of high Intensity interval training versus moderate intensity continuous training on arterial stiffness and 24h blood pressure responses: a systematic review and meta-analysis. J Sci Med Sport 22(4):385–391 30803498 10.1016/j.jsams.2018.09.228

[CR43] Way KL et al (2020) The effect of low volume high intensity interval training on cardiovascular health outcomes in type 2 diabetes: A randomised controlled trial. Int J Cardiol 320(1):148–154 32598997 10.1016/j.ijcard.2020.06.019

[CR44] Westerhof N, Westerhof BE (2013) A review of methods to determine the functional arterial parameters stiffness and resistance. J Hypertens 31(9):1769–1775 23777762 10.1097/HJH.0b013e3283633589

[CR45] Wilkinson IB et al (2000) The influence of heart rate on augmentation index and central arterial pressure in humans. J Physiol 525(Pt 1):263–270 10811742 10.1111/j.1469-7793.2000.t01-1-00263.x PMC2269933

[CR46] Woodman RJ et al (1985) Improved analysis of brachial artery ultrasound using a novel edge-detection software system. J Appl Physiol 91(2):929–937 10.1152/jappl.2001.91.2.929 11457812

