# Supplementary material for: Effect of low-volume combined aerobic and resistance high-intensity interval training on vascular health in people with type 2 diabetes: a randomised controlled trial
Source: Eur J Appl Physiol. 2024 May 2;124(9):2819–33. doi: 10.1007/s00421-024-05473-8 (PMC11365856; doi:10.1007/s00421-024-05473-8)
Supplement: Supplementary file 1 — Supplementary file1 (DOCX 109 KB) [file 421_2024_5473_MOESM1_ESM.docx]

Assessed for eligibility (*n*=445)

Excluded (*n*=376)

- Not meeting inclusion criteria (*n*=166)
- Declined to participate (*n*=76)
- Other reasons (e.g., no response; *n*=134)

Allocated to C-HIIT (*n*=23)

- Received allocated intervention (*n*=21)
- Did not receive allocated intervention (discontinued due to unrelated medical reasons; *n*=2)

**Allocation**

Randomised for the E4D Trial (*n*=69)

**Enrolment**

Allocated to C-MICT (*n*=23)

- Received allocated intervention (*n*=22)
- Did not receive allocated intervention (dropped out prior to program start; *n*=1)

Allocated to Waitlist CON (*n*=23)

- Received allocated intervention (*n*=18)
- Did not receive allocated intervention
  (lost to follow-up; *n*=5)

Total analysed (*n*=23)

Total analysed (*n*=23)

Total analysed (*n*=23)

**Phase 1 Analysis**

**Randomisation**

**Figure S1. CONSORT diagram for Phase 1 intention-to-treat analysis.** C-HIIT (Combined High-Intensity Interval Training); C-MICT (Combined Moderate Intensity Continuous Training); CON (Waitlist Control); E4D (Exercise for Type 2 Diabetes).

Lost to follow-up (unable to contact, dropped out; *n*=6)

Allocated to C-HIIT (*n*=33)

- Received allocated intervention (*n*=30)
- Did not receive allocated intervention (discontinued due to unrelated medical reasons; *n*=3)

**Allocation**

**Phase 2 Analysis AnalysisPhase 1 Analysis**

**Follow-Up**

Randomised for the E4D Trial (*n*=63)

- Randomised at baseline (*n*=46)
- Re-randomised after waitlist CON (*n*=17)

Total analysed (*n*=33)

**Randomisation Enrolment**

Allocated to C-MICT (*n*=30)

- Received allocated intervention (*n*=27)
- Did not receive allocated intervention (dropped out prior to program start; *n*=3)

Lost to follow-up (unable to contact, dropped out; *n*=8)

Total analysed (*n*=29)

- Did not follow pre-testing preparation requirements at follow-up (*n*=1)

Declined re-randomisation from waitlist CON (*n*=1)

**Figure S2. CONSORT diagram for Phase 2 intention-to-treat analysis.** C-HIIT (Combined High-Intensity Interval Training); C-MICT (Combined Moderate Intensity Continuous Training); CON (Waitlist Control); E4D (Exercise for Type 2 Diabetes).

**Table S1. Vascular health outcomes at baseline and after eight weeks of C-HIIT, C-MICT and CON (phase one) – per-protocol (70% attendance and adherence to prescription) analysis**

|  | **C-HIIT** | | | **C-MICT** | | | **CON** | | | **Mean Difference^a^**  **(95% CI) [sample sizes for comparator groups]** | | | | |
| --- | --- | --- | --- | --- | --- | --- | --- | --- | --- | --- | --- | --- | --- | --- |
| **Variable** | **Baseline** | **8 weeks** | **∆** | **Baseline** | **8 weeks** | **∆** | **Baseline** | **8 weeks** | **∆** | **C-HIIT – CON** | **C-MICT – CON** | **C-HIIT – C-MICT** |  |  |
| **Haemodynamic Indices** |  |  |  |  |  |  |  |  |  |  |  |  |  |  |
| Heart rate, bpm | 63 ± 8 | 62 ± 8 | –1 ± 9 | 62 ± 10 | 60 ± 9 | –2 ± 4 | 68 ± 14 | 65 ± 13 | –3 ± 4 | 1.2  (–1.9, 4.3) [19, 18] | 0.4  (–2.1, 2.9) [22, 18] | 0.8  (–1.9, 3.4) [19, 22] |  |  |
| bSBP, mmHg | 132 ± 13 | 132 ± 11 | 0 ± 9 | 131 ± 15 | 129 ± 13 | –2 ± 8 | 132 ± 19 | 129 ± 15 | –3 ± 15 | 1.9  (–5.0, 8.8) [19, 18] | 0.1  (–6.4, 6.7) [22, 18] | 1.7  (–3.2, 6.7) [19, 22] |  |  |
| bDBP, mmHg | 77 ± 6 | 76 ± 7 | –2 ± 5 | 77 ± 10 | 75 ± 9 | –3 ± 6 | 76 ± 10 | 74 ± 9 | –2 ± 10 | 1.2  (–3.5, 6.0) [19, 18] | 0.3  (–4.2, 4.9) [22, 18] | 0.9  (–2.5, 4.3) [19, 22] |  |  |
| MAP, mmHg | 97 ± 7 | 94 ± 7 | –1 ± 6 | 95 ± 11 | 93 ± 9 | –2 ± 6 | 93 ± 11 | 91 ± 8 | –2 ± 12 | 2.3  (–2.6, 7.1) [19, 18] | 0.9  (–3.9, 5.7) [22, 18] | 1.2  (–2.4, 4.8) [19, 22] |  |  |
| cSBP, mmHg | 120 ± 11 | 120 ± 9 | –1 ± 8 | 120 ± 12 | 118 ± 11 | –2 ± 8 | 120 ± 18 | 118 ± 13 | –2 ± 14 | 0.9  (–5.2, 6.9) [19, 18] | –0.6  (–6.3, 5.1) [22, 18] | 1.5  (–3.0, 5.9) [19, 22] |  |  |
| cDBP, mmHg | 78 ± 6 | 77 ± 7 | –1 ± 5 | 79 ± 10 | 76 ± 9 | –3 ± 6 | 77 ± 10 | 75 ± 9 | –2 ± 10 | 1.2  (–3.6, 6.0) [19, 18] | 0.1  (–4.5, 4.7) [22, 18] | 1.1  (–2.3, 4.5) [19, 22] |  |  |
| cPP, mmHg | 42 ± 9 | 43 ± 7 | 1 ± 6 | 41 ± 8 | 42 ± 9 | 1 ± 4 | 43 ± 13 | 43 ± 11 | 1 ± 6 | –0.1  (–3.7, 3.5) [19, 18] | –0.3  (–3.5, 3.0) [22, 18] | 0.3  (–2.7, 3.3) [19, 22] |  |  |
| AIx, % | 28 ± 6 | 27 ± 6 | 0 ± 5 | 27 ± 7 | 26 ± 8 | 0 ± 5 | 25 ± 9 | 27 ± 11 | 2 ± 8 | –1.6  (–6.1, 3.0) [18, 18] | –2.0  (–6.2, 2.2) [22, 18] | 0.3  (–2.8, 3.3) [18, 22] |  |  |
| AIx@75, % | 22 ± 7 | 22 ± 8 | –1 ± 6 | 20 ± 5 | 19 ± 7 | –1 ± 4 | 21 ± 9 | 21 ± 11 | 1 ± 8 | –1.1  (–5.9, 3.8) [18, 18] | –1.9  (–6.0, 2.3) [22, 18] | 0.7  (–2.7, 4.1) [18, 23] |  |  |
| Forward pressure wave, mmHg | 28 ± 6 | 29 ± 6 | 1 ± 5 | 26 ± 5 | 26 ± 6 | 0 ± 3 | 29 ± 7 | 28 ± 6 | –1 ± 4 | 2.0  (–0.8, 4.8) [19, 18] | 0.4  (–1.8, 2.6) [22, 18] | 1.5  (–0.9, 3.9) [19, 22] |  |  |
| Reflected pressure wave, mmHg | 18 ± 4 | 18 ± 3 | 1 ± 3 | 18 ± 4 | 18 ± 4 | 0 ± 2 | 18 ± 6 | 19 ± 6 | 0 ± 3 | 0.0  (–1.9, 1.8) [19, 18] | –0.2  (–1.9, 1.4) [22, 18] | 0.3  (–1.1, 1.6) [19, 22] |  |  |
| Reflection magnitude, % | 63 ± 10 | 63 ± 9 | 0 ± 7 | 70 ± 13 | 71 ± 15 | 1 ± 6 | 64 ± 13 | 68 ± 14 | –4 ± 12 | –5.1  (–11.3, 1.2) [19, 18] | –2.2  (–8.4, 4.0) [22, 18] | –2.5  (–7.0, 2.0) [19, 22] |  |  |
| **Arterial Stiffness** |  |  |  |  |  |  |  |  |  |  |  |  |  |  |
| cfPWV, m·sec^–1^ | 8.4 ± 1.6 | 8.9 ± 1.3 | 0.5 ± 1.3 | 9.3 ± 1.3 | 9.3 ± 1.3 | 0.0 ± 0.6 | 9.9 ± 1.9 | 9.8 ± 1.6 | –0.1 ± 1.3 | –0.1  (–1.0, 0.7) [17, 17] | –0.1  (–0.7, 0.5) [22, 17] | 0.2  (–0.4, 0.8) [17, 22] |  |  |
| **Aortic Reservoir Pressure** |  |  |  |  |  |  |  |  |  |  |  |  |  |  |
| ARP, mmHg | 114 ± 11 | 114 ± 9 | 0 ± 9 | 115 ± 12 | 112 ± 11 | –3 ± 8 | 113 ± 17 | 111 ± 11 | –2 ± 14 | 2.2  (–3.6, 8.0) [19, 18] | 0.0  (–5.6, 5.6) [22, 18] | 2.2  (–2.6, 7.0) [19, 22] |  |  |
| ARP less DBP, mmHg | 34 ± 8 | 35 ± 6 | 1 ± 6 | 33 ± 6 | 33 ± 8 | 0 ± 3 | 34 ± 10 | 34 ± 7 | 0 ± 7 | 1.3  (–2.1, 4.6) [19, 18] | –0.2  (–3.2, 2.8) [22, 18] | 1.4  (–1.7, 4.5) [19, 22] |  |  |
| ARP AUC, mmHg | 10.7 ± 2.3 | 10.9 ± 2.6 | 0.2 ± 2.3 | 9.8 ± 2.4 | 9.5 ± 2.5 | –0.3 ± 1.4 | 10.2 ± 3.3 | 9.8 ± 2.3 | –0.4 ± 2.7 | 0.9  (–0.5, 2.3) [19, 18] | –0.1  (–1.2, 1.1) [22, 18] | 0.8  (–0.5, 2.0) [19, 22] |  |  |
| **Flow–Mediated Dilation** |  |  |  |  |  |  |  |  |  |  |  |  |  |  |
| Resting diameter, mm | 4.0 ± 0.1 | 4.1 ± 0.2 | 0.2 ± 0.4 | 4.5 ± 1.1 | 4.5 ± 1.1 | 0 ± 0.3 | 4.6 ± 0.5 | 4.7 ± 0.8 | 0.1 ± 0.7 | 0.0  (–1.0, 1.0) [10, 13] | –0.1  (–0.6, 0.4) [14, 13] | –0.2  (–0.2, 0.5) [10, 14] |  |  |
| FMD, mm | 0.1 ± 0.1 | 0.2 ± 0.1 | 0.04 ± 0.03 | 0.1 ± 0.1 | 0.2 ± 0.1 | 0.02 ± 0.06 | 0.2 ± 0.1 | 0.1 ± 0.1 | –0.01 ± 0.04 | 0.04  (–0.02, 0.07) [10, 13] | 0.03  (–0.00, 0.07) [14, 13] | 0.01  (–0.03, 0.05) [10, 14] |  |  |
| FMD, % | 3.6 ± 1.8 | 4.3 ± 1.5 | 0.7 ± 0.8 | 3.6 ± 1.6 | 4.0 ± 1.9 | 0.4 ± 1.4 | 3.5 ± 2.0 | 3.2 ± 1.7 | –0.3 ± 0.8 | **1.0***  **(0.2, 1.8) [10, 13]** | 0.8  (–0.2, 1.7) [14, 13] | 0.2  (–0.9, 1.3) [10, 14] |  |  |
| Resting blood flow, ml·s^–1^ | 1.3 ± 0.9 | 1.1 ± 0.6 | –0.2 ± 0.9 | 1.0 ± 0.5 | 1.3 ± 0.9 | 0.4 ± 0.8 | 1.0 ± 0.7 | 1.3 ± 0.9 | 0.3 ± 0.6 | –0.3  (–1.0, 0.3) [10, 13] | –0.1  (–0.5, 0.7) [14, 13] | –0.4  (–1.1, 0.3) [10, 14] |  |  |
| Peak blood flow, ml·s^–1^ | 4.1 ± 1.8 | 5.5 ± 2.3 | 1.4 ± 2.4 | 4.7 ± 2.7 | 5.0 ± 3.9 | 0.6 ± 3.0 | 4.7 ± 3.0 | 5.5 ± 2.3 | 0.7 ± 2.9 | 0.2  (–1.8, 2.2) [10, 13] | –0.1  (–2.5, 2.2) [14, 13] | 0.6  (–2.0, 32) [10, 14] |  |  |
| FMD SR_AUC_, 10^3^·s^–1^ | 17.8 ± 7.6 | 15.9 ± 5.1 | –1.9 ± 6.1 | 12.4 ± 6.1 | 11.6 ± 7.0 | –0.6 ± 7.2 | 12.3 ± 5.3 | 15.0 ± 7.7 | 2.2 ± 6.7 | –1.6  (–7.5, 4.3) [10, 13] | –2.8  (–8.5, 2.9) [14, 13] | 1.7  (–4.0, 7.4) [10, 14] |  |  |
| Time to peak diameter, s | 63 ± 30 | 42 ± 12 | –21 ± 27 | 50 ± 19 | 48 ± 23 | –2 ± 14 | 56 ± 26 | 57 ± 30 | 0 ± 23 | **–17.3***  **(–36.4, –1.7) [10, 13]** | –3.1  (–18.8, 12.6) [14, 13] | –11.7  (–26.7, 3.3) [10, 14] |  |  |
| Data are presented as mean ± standard deviation. *Boldface indicates statistical significance (p≤0.05).  ^a^Mean Difference calculated as difference between change scores for C-HIIT and CON, C-MICT and CON, and C-HIIT and C-MICT after 8 weeks, respectively  **∆** (change score); Aix (augmentation index); Aix@75 (augmentation index adjusted for a heart rate of 75bpm); ARP (aortic reservoir pressure); AUC (area under the curve); bDBP (brachial diastolic blood pressure); bSBP (brachial systolic blood pressure); cDBP (central diastolic blood pressure); cfPWV (carotid-femoral pulse wave velocity); C-HIIT (Combined High-Intensity Interval Training); C-MICT (Combined Moderate Intensity Continuous Training); CON (Waitlist Control); cPP (central pulse pressure); cSBP (central systolic blood pressure); FMD (flow-mediated dilation); MAP (mean arterial pressure); SR_AUC_ (shear rate area under the curve). | | | | | | | | | | | | | |  |

**Table S2. Vascular health outcomes at baseline and after eight weeks of C-HIIT, C-MICT and CON (phase one) – excluding participants with cardiac medication changes**

|  | **C-HIIT** | | | **C-MICT** | | | **CON** | | | **Mean Difference^a^**  **(95% CI) [sample sizes for comparator groups]** | | |
| --- | --- | --- | --- | --- | --- | --- | --- | --- | --- | --- | --- | --- |
| **Variable** | **Baseline** | **8 weeks** | **∆** | **Baseline** | **8 weeks** | **∆** | **Baseline** | **8 weeks** | **∆** | **C-HIIT – CON** | **C-MICT – CON** | **C-HIIT – C-MICT** |
| **Haemodynamic Indices** |  |  |  |  |  |  |  |  |  |  |  |  |
| Heart rate, bpm | 64 ± 8 | 63 ± 8 | –1 ± 5 | 60 ± 9 | 59 ± 9 | –1 ± 4 | 67 ± 13 | 65 ± 13 | –2 ± 4 | 1.3  (–1.2, 3.9) [20, 23] | 0.7  (–1.6, 3.0) [20, 23] | 0.8  (–1.8, 3.4) [20, 20] |
| bSBP, mmHg | 133 ± 13 | 133 ± 10 | 1 ± 9 | 128 ± 11 | 127 ± 10 | –1 ± 8 | 133 ± 17 | 131 ± 14 | –2 ± 14 | 2.6  (–3.2, 8.4) [20, 23] | –0.8  (–6.8, 5.1) [20, 23] | 3.2  (–1.4, 7.7) [20, 20] |
| bDBP, mmHg | 78 ± 6 | 76 ± 6 | –1 ± 5 | 76 ± 10 | 74 ± 9 | –2 ± 6 | 76 ± 9 | 74 ± 8 | –2 ± 9 | 1.7  (–2.3, 5.7) [20, 23] | 0.2  (–4.0, 4.3) [20, 23] | 1.0  (–2.4, 4.4) [20, 20] |
| MAP, mmHg | 96 ± 7 | 95 ± 6 | –1 ± 6 | 93 ± 9 | 92 ± 8 | –2 ± 6 | 94 ± 10 | 92 ± 8 | –2 ± 11 | 2.6  (–1.6, 6.7) [20, 23] | –0.0  (–4.4, 4.3) [20, 23] | 2.0  (–1.5, 5.4) [20, 20] |
| cSBP, mmHg | 120 ± 10 | 121 ± 9 | 1 ± 7 | 118 ± 10 | 117 ± 9 | –1 ± 8 | 120 ± 16 | 119 ± 12 | –1 ± 12 | 1.5  (–3.6, 6.6) [20, 23] | –1.2  (–6.4, 4.0) [20, 23] | 2.6  (–1.6, 6.7) [20, 20] |
| cDBP, mmHg | 79 ± 6 | 78 ± 6 | –1 ± 5 | 77 ± 10 | 75 ± 9 | –2 ± 6 | 77 ± 9 | 75 ± 8 | –2 ± 9 | 1.8  (–2.2, 5.8) [20, 23] | 0.1  (–4.2, 4.3) [20, 23] | 1.4  (–2.0, 4.7) [20, 20] |
| cPP, mmHg | 41 ± 9 | 43 ± 7 | 2 ± 5 | 41 ± 7 | 42 ± 8 | 1 ± 4 | 43 ± 12 | 44 ± 11 | 1 ± 6 | 0.3  (–2.6, 3.2) [20, 23] | –0.6  (–3.6, 2.4) [20, 23] | 1.1  (–1.5, 3.7) [20, 20] |
| AIx, % | 24 ± 10 | 27 ± 7 | 1 ± 4 | 27 ± 7 | 27 ± 8 | –1 ± 5 | 25 ± 10 | 27 ± 11 | 2 ± 7 | –1.3  (–5.2, 2.6) [18, 23] | –2.4  (–6.3, 1.5) [20, 23] | 1.0  (–2.0, 4.0) [18, 20] |
| AIx@75, % | 21 ± 8 | 21 ± 9 | 1 ± 5 | 20 ± 6 | 19 ± 7 | –1 ± 4 | 20 ± 10 | 21 ± 12 | 1 ± 7 | –0.1  (–4.2, 3.9) [18, 23] | –2.0  (–5.8, 1.8) [20, 23] | 1.9  (–1.2, 5.0) [18, 20] |
| Forward pressure wave, mmHg | 28 ± 6 | 30 ± 6 | 2 ± 4 | 25 ± 5 | 25 ± 8 | 0 ± 3 | 30 ± 6 | 29 ± 5 | –1 ± 4 | **2.3***  **(0.1, 4.6) [20, 23]** | 0.1  (–2.0, 2.3) [20, 23] | 2.2  (–0.1, 4.5) [20, 20] |
| Reflected pressure wave, mmHg | 17 ± 3 | 18 ± 3 | 1 ± 2 | 18 ± 3 | 78 ± 3 | 0 ± 2 | 18 ± 6 | 19 ± 5 | 0 ± 3 | 0.4  (–1.1, 1.9) [20, 23] | –0.3  (–1.8, 1.2) [20, 23] | 0.8  (–0.4, 2.0) [20, 20] |
| Reflection magnitude, % | 63 ± 10 | 62 ± 8 | 0 ± 7 | 72 ± 13 | 73 ± 15 | 1 ± 6 | 62 ± 13 | 66 ± 14 | –4 ± 11 | –4.4  (–9.8, 1.0) [20, 23] | –2.1  (–8.1, 3.8) [20, 23] | –2.4  (–7.1, 2.2) [20, 20] |
| **Arterial Stiffness** |  |  |  |  |  |  |  |  |  |  |  |  |
| cfPWV, m·sec^–1^ | 8.6 ± 1.6 | 9.0 ± 1.2 | 0.4 ± 1.3 | 9.1 ± 1.2 | 9.1 ± 1.3 | 0.1 ± 0.6 | 9.6 ± 1.8 | 9.6 ± 1.6 | –0.0 ± 1.2 | –0.0  (–0.7, 0.7) [19, 21] | –0.0  (–0.6, 0.5) [20, 21] | 0.2  (–0.4, 0.7) [19, 20] |
| **Aortic Reservoir Pressure** | |  |  |  |  |  |  |  |  |  |  |  |
| ARP, mmHg | 114 ± 11 | 115 ± 9 | 0 ± 9 | 113 ± 9 | 111 ± 9 | –2 ± 8 | 114 ± 16 | 112 ± 11 | –2 ± 13 | 2.4  (–2.6, 7.5) [20, 23] | –0.9  (–5.9, 4.0) [20, 23] | 3.1  (–1.4, 7.7) [20, 20] |
| ARP less DBP, mmHg | 34 ± 8 | 35 ± 6 | 2 ± 6 | 33 ± 5 | 32 ± 7 | 0 ± 4 | 34 ± 10 | 34 ± 7 | 0 ± 6 | 1.4  (–1.5, 4.3) [20, 23] | –0.8  (–3.5, 1.9) [20, 23] | 2.2  (–0.7, 5.1) [20, 20] |
| ARP AUC, mmHg | 10.4 ± 2.3 | 11.0 ± 2.3 | 0.6 ± 2.1 | 9.4 ± 2.0 | 9.2 ± 2.2 | –0.2 ± 1.5 | 10.3 ± 3.2 | 10.0 ± 2.4 | –0.3 ± 2.4 | 0.9  (–0.2, 2.1) [20, 23] | –0.3  (–1.4, 0.8) [20, 23] | 1.1  (–0.0, 2.2) [20, 20] |
| **Flow–Mediated Dilation** |  |  |  |  |  |  |  |  |  |  |  |  |
| Resting diameter, mm | 4.0 ± 0.7 | 4.2 ± 0.7 | 0.2 ± 0.4 | 4.5 ± 1.1 | 4.5 ± 1.1 | 0 ± 0.3 | 4.6 ± 0.5 | 4.7 ± 0.8 | 0.1 ± 0.7 | 0.0  (–0.5, 0.6) [10, 15] | –0.1  (–0.5, 0.3) [14, 15] | 0.2  (–0.1, 0.5) [10, 14] |
| FMD, mm | 0.2 ± 0.1 | 0.2 ± 0.1 | 0.03 ± 0.02 | 0.2 ± 0.1 | 0.2 ± 0.1 | 0.03 ± 0.06 | 0.2 ± 0.1 | 0.2 ± 0.1 | –0.01 ± 0.04 | **0.04***  **(0.01, 0.06) [10, 15]** | **0.04***  **(0.00, 0.08) [14, 15]** | 0.00  (–0.05, 0.04) [10, 14] |
| FMD, % | 3.9 ± 1.6 | 4.4 ± 1.4 | 0.5 ± 0.7 | 3.8 ± 2.0 | 4.5 ± 2.6 | 0.7 ± 1.4 | 3.8 ± 2.1 | 3.5 ± 1.8 | –0.3 ± 0.8 | **0.7***  **(0.0, 1.4) [10, 15]** | **1.0***  **(0.1, 1.8) [14, 15]** | –0.4  (–1.5, 0.7) [10, 14] |
| Resting blood flow, ml·s^–1^ | 1.1 ± 0.8 | 1.3 ± 0.7 | 0.2 ± 0.9 | 1.0 ± 0.5 | 1.3 ± 0.9 | 0.4 ± 0.8 | 1.1 ± 0.6 | 1.4 ± 0.9 | 0.4 ± 0.7 | -0.2  (–0.8, 0.4) [10, 15] | –0.0  (–0.6, 0.6) [14, 15] | –0.1  (–0.8, 0.6) [10, 14] |
| Peak blood flow, ml·s^–1^ | 3.8 ± 1.8 | 5.5 ± 2.2 | 1.7 ± 2.4 | 4.6 ± 2.6 | 5.2 ± 3.8 | 0.6 ± 3.0 | 5.0 ± 2.9 | 5.9 ± 2.4 | 0.9 ± 2.9 | 0.1  (–1.9, 2.1) [10, 15] | –0.5  (–2.6, 1.7) [14, 15] | 1.0  (–1.5, 3.4) [10, 14] |
| FMD SR_AUC_, 10^3^·s^–1^ | 15.2 ± 7.7 | 15.1 ± 6.2 | –0.1 ± 3.2 | 12.1 ± 6.0 | 11.5 ± 7.0 | –0.6 ± 7.2 | 13.4 ± 5.7 | 16.2 ± 8.3 | 2.9 ± 6.6 | –2.6  (–0.4, 2.1) [10, 15] | –3.8  (–9.0, 1.5) [14, 15] | 1.6  (–3.2, 6.4) [10, 14] |
| Time to peak diameter, s | 54 ± 24 | 39 ± 13 | –15 ± 24 | 48 ± 22 | 46 ± 25 | –2 ± 14 | 56 ± 24 | 567± 28 | 1 ± 22 | –16.8  (–34.1, 0.5) [10, 15] | –4.1  (–18.7, 10.5) [14, 15] | –10.5  (–25.0, 4.1) [10, 14] |
| Data are presented as mean ± standard deviation. *Boldface indicates statistical significance (p≤0.05).  ^a^Mean Difference calculated as difference between change scores for C-HIIT and CON, C-MICT and CON, and C-HIIT and C-MICT after 8 weeks, respectively  **∆** (change score); AIx (augmentation index); AIx@75 (augmentation index adjusted for a heart rate of 75bpm); ARP (aortic reservoir pressure); AUC (area under the curve); bDBP (brachial diastolic blood pressure); bSBP (brachial systolic blood pressure); cDBP (central diastolic blood pressure); cfPWV (carotid-femoral pulse wave velocity); C-HIIT (Combined High-Intensity Interval Training); C-MICT (Combined Moderate Intensity Continuous Training); CON (Waitlist Control); cPP (central pulse pressure); cSBP (central systolic blood pressure); FMD (flow-mediated dilation); MAP (mean arterial pressure); SR_AUC_ (shear rate area under the curve). | | | | | | | | | | | | |

**Table S3. Vascular health outcomes at baseline and after 12 months of C-HIIT and C-MICT, with CON participants re-randomised (phase two) – per-protocol (50% attendance and adherence to prescription) analysis**

|  | **C-HIIT** | | | | | **C-MICT** | | | | | **Mean Time Difference^a^**  **(95% CI) [sample sizes for comparator groups]** | ***p*–value, Time** | ***p*–value, Group x Time** |
| --- | --- | --- | --- | --- | --- | --- | --- | --- | --- | --- | --- | --- | --- |
|  | **Baseline** | **12 months** | | | **∆** | **Baseline** | | **12 months** | | **∆** |  |  |  |
| **Haemodynamic Indices** |  | |  |  | |  |  | |  | |  |  |  |
| Heart rate, bpm | 66 ± 12 | | 66 ± 15 | 0 ± 6 | | 61 ± 8 | 60 ± 10 | | –1 ± 6 | | –0.5  (–5.0, 4.0) [4, 46] | 0.802 | 0.449 |
| bSBP, mmHg | 126 ± 1 | | 121 ± 9 | –4 ± 11 | | 137 ± 11 | 126 ± 10 | | –10 ± 11 | | –7.3  (–15.5, 0.9) [4, 6] | 0.073 | 0.411 |
| bDBP, mmHg | 74 ± 5 | | 69 ± 8 | –5 ± 8 | | 78 ± 10 | 71 ± 6 | | –7 ± 8 | | –5.5  (–11.4, 0.4) [4, 6] | 0.063 | 0.705 |
| MAP, mmHg | 91 ± 3 | | 87 ± 7 | –4 ± 9 | | 97 ± 10 | 89 ± 7 | | –8 ± 9 | | –6.1  (–12.5, 0.3) [4, 6] | 0.059 | 0.539 |
| cSBP, mmHg | 114 ± 3 | | 109 ± 9 | –5 ± 10 | | 124 ± 10 | 114 ± 8 | | –10 ± 10 | | **–7.4***  **(–14.6, –0.1) [4, 6]** | **0.047*** | 0.471 |
| cDBP, mmHg | 75 ± 4 | | 70 ± 8 | –5 ± 8 | | 79 ± 10 | 72 ± 6 | | –7 ± 8 | | –5.8  (–11.9, 0.4) [4, 6] | 0.062 | 0.774 |
| cPP, mmHg | 39 ± 3 | | 39 ± 5 | 0 ± 4 | | 45 ± 2 | 42 ± 3 | | –3 ± 4 | | –1.6  (–4.8, 1.7) [4, 6] | 0.291 | 0.921 |
| AIx, % | 29 ± 7 | | 27 ± 11 | –2 ± 7 | | 28 ± 6 | 25 ± 9 | | –3 ± 7 | | –2.6  (–7.9, 2.8) [4, 6] | 0.301 | 0.774 |
| AIx@75, % | 24 ± 7 | | 23 ± 16 | –2 ± 9 | | 21 ± 8 | 17 ± 11 | | –4 ± 9 | | –2.8  (–9.8, 4.2) [4, 6] | 0.380 | 0.766 |
| Forward pressure wave, mmHg | 27 ± 2 | | 29 ± 3 | 2 ± 5 | | 29 ± 5 | 27 ± 5 | | –2 ± 5 | | 0.1  (–3.6, 3.8) [4, 6] | 0.957 | 0.202 |
| Reflected pressure wave, mmHg | 17 ± 1 | | 16 ± 3 | –1 ± 2 | | 19 ± 2 | 17 ± 2 | | –2 ± 2 | | **–1.6***  **(–3.2, –0.1) [4, 6]** | **0.045*** | 0.438 |
| Reflection magnitude, % | 65 ± 6 | | 56 ± 8 | –9 ± 1 | | 67 ± 10 | 64 ± 17 | | –2 ± 11 | | –5.4  (–14.4, 3.6) [4, 6] | 0.207 | 0.426 |
|  |  | |  |  | |  |  | |  | |  |  |  |
| **Arterial Stiffness** |  | |  |  | |  |  | |  | |  |  |  |
| cfPWV, m·sec^–1^ | 8.9 ± 1.2 | | 8.7 ± 1.4 | –0.2 ± 0.5 | | 10.3 ± 1.2 | 9.3 ± 1.5 | | –0.8 ± 0.5 | | –0.5  (–1.1, 0.1) [4, 6] | 0.068 | 0.216 |
| **Aortic Reservoir Pressure** |  | |  |  | |  |  | |  | |  |  |  |
| ARP, mmHg | 107 ± 5 | | 105 ± 10 | –2 ± 6 | | 118 ± 10 | 105 ± 9 | | –13 ± 5 | | **–7.5***  **(–14.1, 0.9) [4, 6]** | **0.031*** | 0.089 |
| ARP less DBP, mmHg | 31 ± 4 | | 32 ± 8 | 1 ± 4 | | 36 ± 3 | 32 ± 3 | | –4 ± 3 | | –1.4  (–5.1, 2.4) [4, 6] | 0.422 | 0.197 |
| ARP AUC, mmHg | 10.0 ± 1.4 | | 10.1 ± 2.7 | 0.1 ± 1.5 | | 10.6 ± 2.5 | 9.2 ± 1.4 | | –1.4 ± 1.2 | | –0.7  (–2.2, 0.9) [4, 6] | 0.342 | 0.291 |
| Data are presented as mean ± standard deviation. *Boldface indicates statistical significance (p≤0.05).  ^a^Mean Time Difference calculated as 12 months minus baseline (pooled effects of exercise).  FMD available in *n*=3 only (C-HIIT *n*=1; C-MICT *n*=2), so these outcomes were not included in this analysis  ∆ (change score); AIx (augmentation index); AIx@75 (augmentation index adjusted for a heart rate of 75bpm); ARP (aortic reservoir pressure); AUC (area under the curve); bDBP (brachial diastolic blood pressure); bSBP (brachial systolic blood pressure); cDBP (central diastolic blood pressure); cfPWV (carotid-femoral pulse wave velocity); C-HIIT (Combined High-Intensity Interval Training); C-MICT (Combined Moderate Intensity Continuous Training); CON (Waitlist Control); cPP (central pulse pressure); cSBP (central systolic blood pressure); FMD (flow-mediated dilation); MAP (mean arterial pressure); SRAUC (shear rate area under the curve). | | | | | | | | | | | | | |

**Table S4. Vascular health outcomes at baseline and after 12 months of C-HIIT and C-MICT, with CON participants re-randomised (phase two) – excluding participants with cardiac medication changes**

|  | **C-HIIT** | | | | | **C-MICT** | | | | | **Mean Time Difference^a^**  **(95% CI) [sample sizes for comparator groups]** | ***p*–value, Time** | ***p*–value, Group x Time** |  |
| --- | --- | --- | --- | --- | --- | --- | --- | --- | --- | --- | --- | --- | --- | --- |
|  | **Baseline** | | **12 months** | | **∆** | **Baseline** | | **12 months** | **∆** | |  |  |  |  |
| **Haemodynamic Indices** |  |  | |  | |  |  | | |  |  |  |  |  |
| Heart rate, bpm | 64 ± 10 | 64 ± 10 | | 0 ± 6 | | 62 ± 10 | 61 ± 10 | | | –1 ± 6 | 0.4  (–1.6, 2.4) [22, 19] | 0.699 | 0.738 |  |
| bSBP, mmHg | 131 ± 13 | 129 ± 14 | | –2 ± 10 | | 127 ± 13 | 125 ± 14 | | | –2 ± 10 | –2.0  (–5.4, 1.3) [22, 19] | 0.229 | 0.828 |  |
| bDBP, mmHg | 75 ± 8 | 73 ± 8 | | –2 ± 5 | | 76 ± 8 | 73 ± 8 | | | –2 ± 5 | –**2.1***  (–**3.6,** –**0.6) [22, 19]** | **0.007*** | 0.924 |  |
| MAP, mmHg | 94 ± 8 | 92 ± 9 | | –2 ± 6 | | 93 ± 8 | 91 ± 9 | | | –2 ± 6 | –2.0  (–4.0, 0.1) [22, 19] | 0.056 | 0.947 |  |
| cSBP, mmHg | 119 ± 11 | 118 ± 12 | | –1 ± 9 | | 116 ± 11 | 114 ± 12 | | | –3 ± 9 | –1.9  (–4.8, 1.1) [22, 19] | 0.202 | 0.633 |  |
| cDBP, mmHg | 76 ± 8 | 74 ± 9 | | –2 ± 5 | | 77 ± 8 | 75 ± 9 | | | –2 ± 5 | –**2.2***  **(**–**3.7,** –**0.7) [22, 19]** | **0.006*** | 0.771 |  |
| cPP, mmHg | 43 ± 10 | 44 ± 11 | | 1 ± 7 | | 40 ± 10 | 39 ± 11 | | | –1 ± 7 | 0.3  (–1.9, 2.4) [22, 19] | 0.792 | 0.368 |  |
| AIx, % | 28 ± 9 | 31 ± 11 | | 3 ± 9 | | 24 ± 9 | 23 ± 11 | | | –1 ± 8 | 1.2  (–1.5, 4.0) [20, 19] | 0.353 | 0.119 |  |
| AIx@75, % | 23 ± 10 | 26 ± 11 | | 4 ± 10 | | 18 ± 10 | 17 ± 11 | | | –1 ± 9 | 1.7  (–1.4, 4.8) [20, 19] | 0.267 | 0.138 |  |
| Forward pressure wave, mmHg | 27 ± 5 | 29 ± 6 | | 2 ± 5 | | 25 ± 5 | 27 ± 6 | | | 2 ± 5 | **2.0***  **(0.4, 3.6) [21, 19]** | **0.016*** | 0.914 |  |
| Reflected pressure wave, mmHg | 18 ± 4 | 18 ± 4 | | 0 ± 3 | | 17 ± 4 | 16 ± 4 | | | –1 ± 3 | –0.2  (–1.3, 0.9) [21, 19] | 0.723 | 0.480 |  |
| Reflection magnitude, % | 65 ± 12 | 62 ± 14 | | –3 ± 12 | | 69 ± 12 | 61 ± 15 | | | –8 ± 13 | –**5.6***  **(**–**9.5,** –**1.7) [21, 19]** | **0.006*** | 0.170 |  |
|  |  |  | |  | |  |  | | |  |  |  |  |  |
| **Arterial Stiffness** |  |  | |  | |  |  | | |  |  |  |  |  |
| cfPWV, m·sec^–1^ | 9.2 ± 1.4 | 9.7 ± 1.7 | | 0.5 ± 1.5 | | 9.2 ± 1.4 | 9.1 ± 1.6 | | | –0.1 ± 1.4 | 0.2  (–0.3, 0.7) [20, 19] | 0.341 | 0.494 |  |
| **Aortic Reservoir Pressure** | |  | |  | |  |  | | |  |  |  |  |  |
| ARP, mmHg | 112 ± 10 | 110 ± 11 | | –3 ± 8 | | 111 ± 10 | 104 ± 11 | | | –7 ± 9 | –**4.8***  **(**–**7.5, –2.1) [22, 19]** | **0.001*** | 0.169 |  |
| ARP less DBP, mmHg | 34 ± 8 | 36 ± 9 | | 2 ± 7 | | 31 ± 8 | 30 ± 9 | | | –1 ± 7 | 0.6  (–1.5, 2.6) [22, 19] | 0.585 | 0.155 |  |
| ARP AUC, mmHg | 10.3 ± 2.4 | 10.7 ± 2.7 | | 0.4 ± 1.9 | | 8.9 ± 2.4 | 8.6 ± 2.7 | | | –0.3 ± 1.9 | 0.1  (–0.6, 0.7) [22, 19] | 0.848 | 0.207 |  |
| **Flow–Mediated Dilation** |  |  | |  | |  |  | | |  |  |  |  |  |
| Resting diameter, mm | 4.3 ± 1.0 | 4.1 ± 1.1 | | –0.2 ± 1.0 | | 4.5 ± 1.0 | 4.4 ± 1.4 | | | –0.1 ± 1.0 | –0.1  (–0.3, 0.6) [13, 13] | 0.501 | 0.768 |  |
| FMD, mm | 0.2 ± 0.1 | 0.2 ± 0.1 | | 0.04 ± 0.12 | | 0.2 ± 0.1 | 0.2 ± 0.1 | | | –0.02 ± 0.04 | 0.01  (–0.03, 0.05) [13, 13] | 0.608 | 0.147 |  |
| FMD, % | 3.8 ± 2.0 | 4.8 ± 2.3 | | 1.0 ± 2.1 | | 4.1 ± 2.0 | 3.7 ± 3.0 | | | –0.3 ± 3.0 | 0.3  (–0.8, 1.4) [13, 13] | 0.525 | 0.206 |  |
| Resting blood flow, ml·s^–1^ | 1.5 ± 0.9 | 1.2 ± 0.5 | | –0.2 ± 1.3 | | 1.0 ± 0.9 | 1.1 ± 0.5 | | | 0.1 ± 1.8 | -0.1  (–0.7, 0.6) [13, 13] | 0.829 | 0.609 |  |
| Peak blood flow, ml·s^–1^ | 5.2 ± 2.6 | 5.0 ± 3.1 | | –0.2 ± 3.2 | | 4.6 ± 2.6 | 5.6 ± 4.4 | | | 1.0 ± 4.5 | 0.4  (–1.2, 2.1) [13, 13] | 0.598 | 0.441 |  |
| FMD SR_AUC_, 10^3^·s^–1^ | 16.5 ± 6.9 | 19.8 ± 7.6 | | 3.3 ± 6.3 | | 11.6 ± 6.9 | 12.6 ± 10.1 | | | 10.0 ± 10.1 | 2.1  (–1.2, 5.4) [13, 13] | 0.189 | 0.476 |  |
| Time to peak diameter, s | 53 ± 25 | 69 ± 29 | | 16 ± 32 | | 49 ± 25 | 38 ± 42 | | | –11 ± 44 | 2.6  (–13.2, 18.4) [13, 13] | 0.730 | 0.091 |  |
| Data are presented as mean ± standard deviation. *Boldface indicates statistical significance (p≤0.05).  ^a^Mean Time Difference calculated as 12 months minus baseline (pooled effects of exercise).  ∆ (change score); AIx (augmentation index); AIx@75 (augmentation index adjusted for a heart rate of 75bpm); ARP (aortic reservoir pressure); AUC (area under the curve); bDBP (brachial diastolic blood pressure); bSBP (brachial systolic blood pressure); cDBP (central diastolic blood pressure); cfPWV (carotid-femoral pulse wave velocity); C-HIIT (Combined High-Intensity Interval Training); C-MICT (Combined Moderate Intensity Continuous Training); CON (Waitlist Control); cPP (central pulse pressure); cSBP (central systolic blood pressure); FMD (flow-mediated dilation); MAP (mean arterial pressure); SRAUC (shear rate area under the curve). | | | | | | | | | | | | | | |
